# Convergent Evolution of Mucosal Immune Responses at the Buccal Cavity of Teleost Fish

**DOI:** 10.1016/j.isci.2019.08.034

**Published:** 2019-08-24

**Authors:** Yong-Yao Yu, Wei-Guang Kong, Hao-Yue Xu, Zhen-Yu Huang, Xiao-Ting Zhang, Li-Guo Ding, Shuai Dong, Guang-Mei Yin, Fen Dong, Wei Yu, Jia-Feng Cao, Kai-Feng Meng, Xia Liu, Yu Fu, Xue-zhen Zhang, Yong-an Zhang, J. Oriol Sunyer, Zhen Xu

**Affiliations:** 1 Department of Aquatic Animal Medicine, College of Fisheries, Huazhong Agricultural University, Wuhan, Hubei 430070, China; 2 Department of Pathobiology, School of Veterinary Medicine, University of Pennsylvania, Philadelphia, PA 19104, USA; 3 Laboratory for Marine Biology and Biotechnology, Qingdao National Laboratory for Marine Science and Technology, Qingdao, Shandong 266071, China

**Keywords:** Biological Sciences, Immunology, Omics, Transcriptomics

## Abstract

The buccal mucosa (BM) is a critical first line of defense in terrestrial animals. To gain further insights into the evolutionary origins and primordial roles of BM in teleosts here we show that rainbow trout, a teleost fish, contains a diffuse mucosal associated lymphoid tissue (MALT) within its buccal cavity. Upon parasite infection, a fish immunoglobulin specialized in mucosal immunity (sIgT) was induced to a high degree, and parasite-specific sIgT responses were mainly detected in the buccal mucus. Moreover, we show that the trout buccal microbiota is prevalently coated with sIgT. Overall our findings revealed that the MALT is present in the BM of a non-tetrapod species. As fish IgT and mucus-producing cells are evolutionarily unrelated to mammalian IgA and salivary glands, respectively, our findings indicate that mucosal immune responses in the BM of teleost fish and tetrapods evolved through a process of convergent evolution.

## Highlights

• A mucosal associated lymphoid tissue is identified in teleost buccal cavity
• Local immune responses can be induced in teleost buccal mucosa
• Teleost IgT plays important roles in **defending against** buccal pathogen
• Microbiota in teleost buccal mucosa is prevalently coated with secreted IgT

## Graphical Abstract

**Figure:**
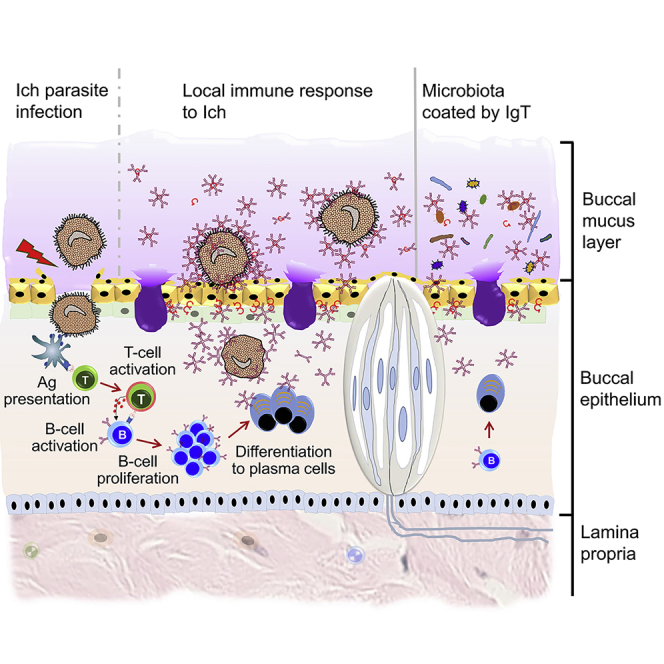

## Introduction

The buccal cavity (BC) of vertebrates is the gateway for both the gastrointestinal and respiratory tracts and is considered a critical mucosal surface in tetrapod species (Winning and Townsend, 2000, Squier and Kremer, 2001, Abbate et al., 2006). Microbes from air, water, and food pose continuous challenges to the homeostasis of the BC (Walker, 2004), and thus, vertebrates have evolved efficient innate and adaptive immune strategies to protect this critical surface. In tetrapod species, secretory IgA (sIgA) is the main humoral component involved in adaptive immune responses against oral pathogens (Brandtzaeg, 2013). Moreover, orally produced sIgA is also involved in the control and homeostasis of the buccal microbiota. sIgA is the most abundant immunoglobulin class in the saliva of mammals (Marcotte and Lavoie, 1998). It is worth noting that saliva is produced only by mammals, birds, and reptiles, and it is in mammals where it is known to have a very important digestive function (Pedersen et al., 2002, Dawes et al., 2015), whereas in birds and reptiles this role is significantly less marked. Interestingly, amphibians are known to contain both mucus-producing cells as well as intermaxillary salivary glands (Latney and Clayton, 2014), although the digestive and adaptive immune roles of their putative saliva and mucosal secretions have been ill investigated. In contrast to all tetrapod species, teleost fish lack salivary glands in their BC, which is instead populated with abundant mucus-secreting cells that produce the mucus that coats their buccal epithelium (Yashpal et al., 2007).

In mammals, some mucosal regions within the BC are covered by a keratinized stratified epithelium (gingival, hard palate, outer lips), whereas other areas, including the ventral side of the tongue, the floor of the mouth, the inner surface of the lips, and cheeks, are lined by a non-keratinized stratified epithelium (Squier and Kremer, 2001). In contrast, the entire buccal epithelium of fish is non-keratinized. Interestingly, the non-keratinized buccal areas of mammals resemble the overall structure of the fish buccal mucosa (BM) as both contain two main layers, an outer layer of stratified squamous epithelium and an underlying layer of dense connective tissue (lamina propria) (Winning and Townsend, 2000, Squier and Kremer, 2001, Abbate et al., 2006). Mammalian sIgA found in the saliva is produced by plasma cells (PCs) localized around the salivary glands (Brandtzaeg, 2013). Upon secretion by PCs, sIgA is actively transported by the polymeric immunoglobulin receptor (pIgR) expressed by parenchymal cells within these glands (Carpenter et al., 2004, Brandtzaeg, 2013). In mammals, the salivary gland within BM is considered a mucosal effector site where IgA-producing plasma cells are derived from mucosal inductive sites localized in the nasopharynx-associated lymphoid tissue and gut-associated lymphoid tissue (i.e., tonsils and Peyer patches) (Jackson et al., 1981, Brandtzaeg, 2007). Whether non-tetrapod species have evolved mucosal adaptive immune responses in the BM is at this point unknown. Since many aquatic environments harbor much higher concentrations of microbes than that found in air, it is reasonable to hypothesize that fish must have evolved an effective mucosal immune system to protect their BC.

Within non-tetrapods, bony and cartilaginous fish represent the earliest vertebrates containing immunoglobulin (Ig). In contrast to mammals that contain five major Ig classes, only three Ig isotypes have been identified in teleosts, IgM, IgD, and IgT/IgZ. IgM is the best characterized teleost Ig isotype both at the molecular and functional levels, and it is the most abundant Ig class in plasma (Salinas et al., 2011). Moreover, IgM represents the prevalent Ig in systemic immune responses (Salinas et al., 2011). Like IgM, IgD is an ancient Ig class that has been found in most jawed vertebrates (Ramirez-Gomez et al., 2012). However, the immune function of fish IgD remains unknown, although secreted IgD has been found coating a small portion of the fish microbiota (Xu et al., 2016) and may function as an innate pattern recognition molecule (Edholm et al., 2010). IgT (also called IgZ in some species) has been described in all studied teleost fish except for medaka and catfish (Danilova et al., 2005, Hansen et al., 2005, Fillatreau et al., 2013). We have previously shown that IgT plays a major role in teleost mucosal immunity, akin to that of IgA tetrapods (Zhang et al., 2010, Ramsey et al., 2010). This discovery broke the old paradigm that mucosal immunoglobulins were present only in tetrapod species. More specifically, we have demonstrated that, upon infection, IgT is the main Ig induced in several mucosal surfaces, including the gut, gills, nose, and skin (Zhang et al., 2010, Xu et al., 2013, Xu et al., 2016, Tacchi et al., 2014, Yu et al., 2018). Significantly, we also found that similar to the role of sIgA in mammals, sIgT is the prevalent Ig coating the microbiota in all fish mucosal areas (Zhang et al., 2010, Tacchi et al., 2014, Xu et al., 2013, Xu et al., 2016).

To gain further insights into the evolutionary origins and primordial roles of buccal adaptive immune responses in vertebrates, here, we investigated the presence and immune roles of a buccal MALT in the BC of a teleost fish, the rainbow trout (*Oncorhynchus mykiss*). Our findings reveal a well-defined diffuse MALT in the trout's BC, and we demonstrate its key role in inducing strong local innate and adaptive immune responses upon infection with *Ichthyophthirius multifiliis* (Ich) parasite. Furthermore, we show that, in addition to being the prevalent local Ig induced upon infection, sIgT is also the main sIg recognizing and coating the trout buccal microbiota. Overall, our findings indicate the presence of a bona fide MALT in the BC of a non-tetrapod species as well as its involvement in both the control of pathogens and recognition of microbiota.

## Results

### Teleost BM Shares the Typical Features of a MALT

**Figure 1:**
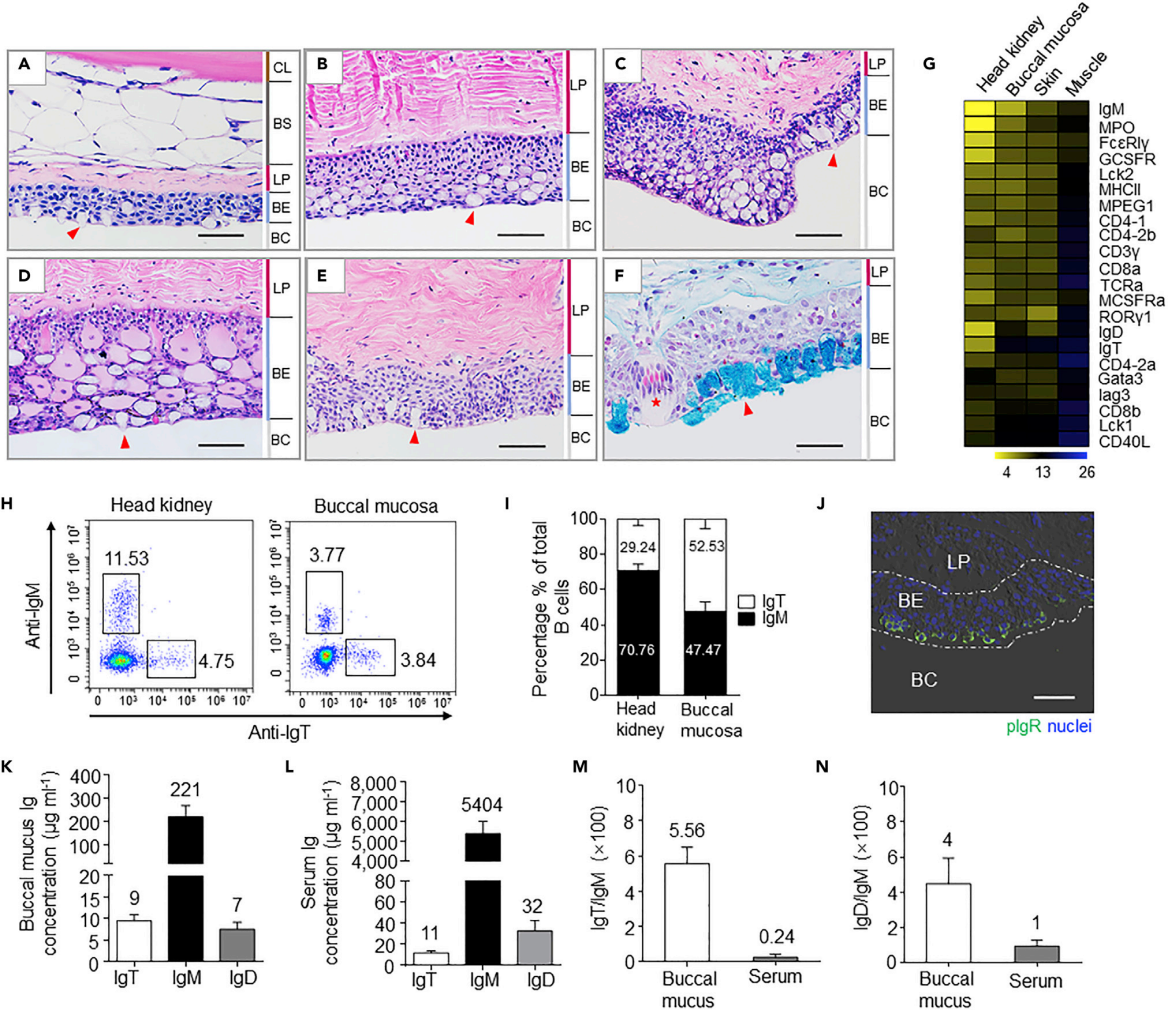
General Organization of Teleost BM (A–E) Hematoxylin and eosin stains of BMs obtained from five different teleost families, including rainbow trout (*Oncorhynchus mykiss*) (A), Japanese sea bass (*Lateolabrax japonicus*) (B), Asian swamp eel (*Monopterus albus*) (C), Southern catfish (*Silurus meridionalis*), (D) and Snakehead (*Channa argus*) (E). BC, buccal cavity; BE, buccal epithelium; LP, lamina propria; BS, buccal submucosa; CL, cartilage layer. Scale bar, 50 μm. (F) AB stain of the BM of a control adult rainbow trout (*Oncorhynchus mykiss*). Black triangles indicate lymphocytes. Red arrows indicate mucous cells. The red asterisk denotes taste bud in BM. BC, buccal cavity; BE, buccal epithelium; LP, lamina propria. Scale bar, 50 μm. (G) Heatmap illustrates results from quantitative real-time PCR of mRNAs for selected immune markers in trout head kidney, BM, skin, and muscle (n = 6). Data are expressed as mean Ct values ±SEM. (H) Flow cytometry analysis of head kidney (Left) and BM (Right) leukocytes stained with anti-IgM and anti-IgT antibodies. Numbers in outlined boxes indicate the percentage of IgM^+^ (Top Left) and IgT^+^ (Bottom Right) B cells in the lymphocyte gate, respectively. (I) Frequency (Mean ± SEM) of IgM^+^ and IgT^+^ B cells among total B cells present in trout head kidney and BM (n = 12). (J) Immunofluorescence staining for pIgR (green) in a paraffinic section of trout BM (n = 9). Nuclei are stained with DAPI (blue) (isotype-matched control antibody staining is shown in Figure S4A). Scale bar, 50 μm. (K and L) Immunoblot and densitometric analysis of the concentration of IgT, IgM, and IgD in buccal mucus (K) and serum (L) (n = 12). (M and N) Ratio of IgT to IgM concentration (M) and IgD to IgM concentration (N) in buccal mucus and serum (n = 12). Data in K–N are representative of at least three independent experiments (Mean ± SEM).

To understand the histological organization of teleost BM (Figures S1A–S1D), paraffin sections of BMs obtained from five different families (Figure S2), Salmonidae, Percichthyidae, Synbranchidae, Siluridae, and Channidae, were stained with both hematoxylin and eosin (H&E) (Figures 1A–1E) and Alcian blue (AB) (Figures 1F and S3A–S3D). We observed that the BM of Japanese sea bass (*Lateolabrax japonicus*), Asian swamp eel (*Monopterus albus*), Southern catfish (*Silurus meridionalis*), and Snakehead (*Channa argus*) contained intraepithelial lymphocytes and lamina propria leukocytes (Figures 1A–1E), with a large number of mucus-producing cells in the buccal epithelium (Figures 1F and S3A–S3D). These results of distribution and structure in the BMs from all five species resemble those of other mucosal tissues. Moreover, using reverse transcription quantitative real-time PCR (qPCR), we measured the levels of expression of gene markers for the main myeloid and lymphoid cell types in the BM from the control adult rainbow trout. We then compared them to those in the head kidney, skin, and muscle. We found that consistently high levels of expression of most immune markers were detected in the BM, head kidney, and skin, indicating an unrecognized immunological function of the BM in rainbow trout (Figure 1G). The abundance of two main B cell subsets (IgM^+^ and IgT^+^ B cells) in the BM was analyzed by flow cytometry (Figure 1H). We found that, similar to the gut, skin, gills, and nose (Zhang et al., 2010, Xu et al., 2013, Xu et al., 2016, Tacchi et al., 2014), IgT^+^ B cells make up ∼52.53% of the total B cells in the BM of rainbow trout, whereas ∼47.47% of the total B cells are IgM^+^ (Figure 1I). In contrast, only ∼29.24% of IgT^+^ B cells were detected in the head kidney (Figure 1I). Mucosal Igs have been previously reported to be transported across the mucosal epithelium via polymeric Ig receptors (pIgRs). Here, using a polyclonal anti-trout pIgR antibody, a large portion of the epithelial cells of the BM were stained by immunofluorescence and found to be located in the apical areas of the mucosal epithelium of the trout (Figure 1J; isotype-matched control antibodies, Figure S4A). Next, we analyzed the concentration of IgT, IgM, and IgD in the buccal mucus and serum by western blotting. We found that, although the protein concentration of IgT was ∼23- and ∼453-fold lower than that of IgM in buccal mucus and serum, respectively (Figures 1K and 1L), the ratio of IgT/IgM in the buccal mucus was ∼23-fold greater than that in the serum (Figure 1M). Interestingly, the concentration of IgD did not differ significantly from that of IgT in the buccal mucus, whereas in the serum, the concentration of IgD was ∼3-fold higher than that of IgT and ∼165-fold lower than that of IgM (Figures 1K and 1L). The ratio of IgD/IgM was ∼5-fold higher in the buccal mucus than in the serum (Figure 1N). To understand whether the different trout immunoglobulins were in monomeric or polymeric form in the buccal mucus, we collected and processed buccal mucus of rainbow trout and loaded it into a gel filtration column. A large portion of IgT in the buccal mucus was found in polymeric form, as it eluted at a fraction similar to that of trout IgM, a tetrameric Ig (Figure S5A). In contrast, a small portion of IgT in the buccal mucus was eluted in monomeric form. Interestingly, by SDS-PAGE under non-reducing conditions, polymeric IgT (pIgT) in buccal mucus migrated in the same position as monomeric IgT, indicating that pIgT subunits are associated by non-covalent interactions (Figure S5B, left). However, IgM and IgD in the buccal mucus migrated as a polymer and a monomer, respectively (Figure S5B, right and middle), similar to the finding previously reported by us in the gill mucus (Xu et al., 2016).

### Trout Buccal Bacteria Are Coated by Mucosal Igs

**Figure 2:**
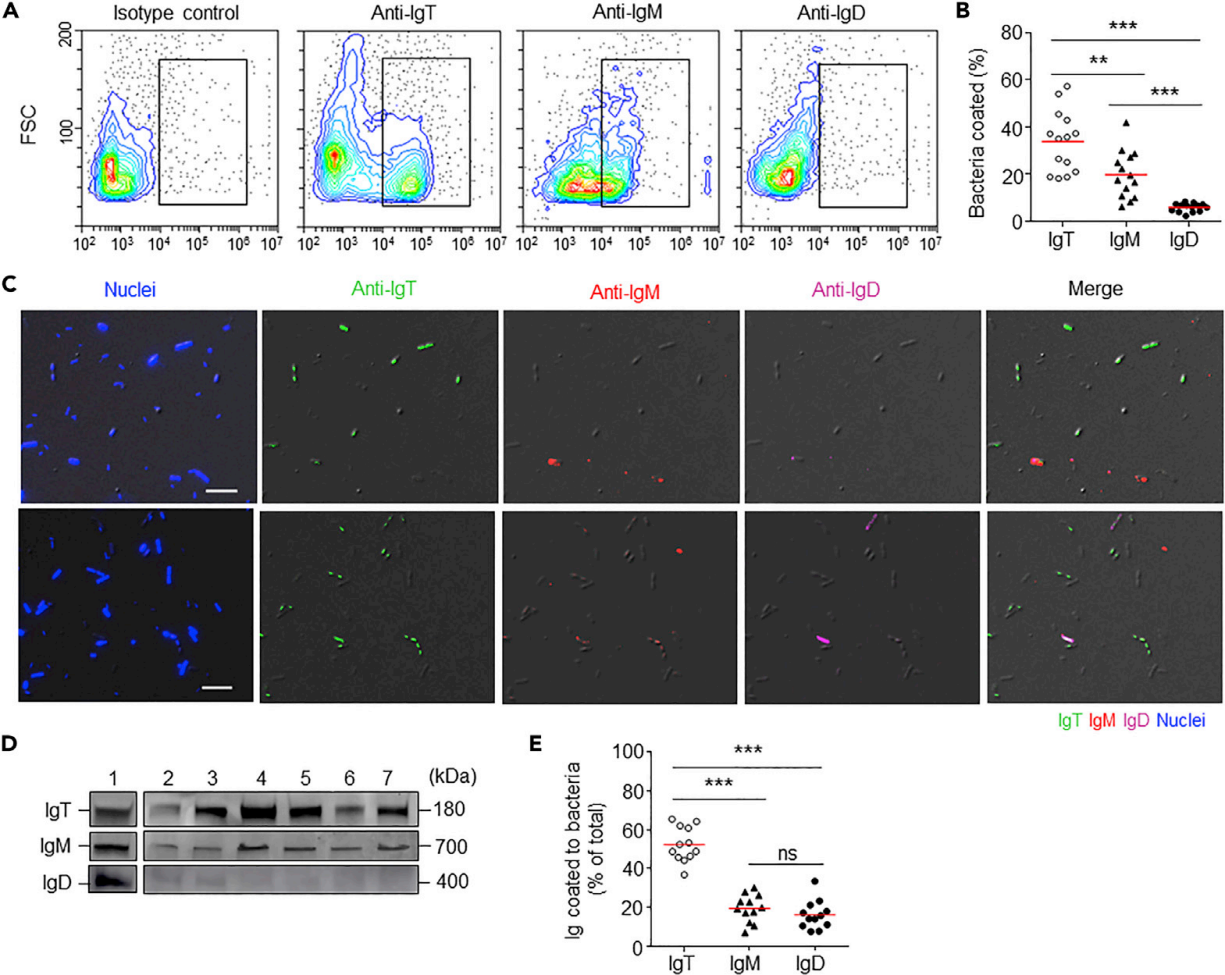
Trout Buccal Bacteria Are Predominantly Coated with IgT (A) Representative scatterplots showing the staining of buccal bacteria with IgT, IgM, and IgD. Bacteria were stained with isotype controls, anti-trout IgT, or anti-trout IgM or anti-trout IgD mAbs, respectively. (B) Percentage of buccal bacteria coated with IgT, IgM, or IgD (n = 14). The median percentage is shown by a red line. Statistical differences between the percentage of buccal bacteria coated with IgT or IgM or IgD were evaluated by one-way ANOVA with Bonferroni correction. (C) Differential interference contrast (DIC) images of buccal bacteria stained with a DAPI-Hoeschst solution (blue), anti-IgT (green), anti-IgM (red), or anti-IgD (magenta), and merging IgT, IgM, and IgD stainings (merge). (Isotype-matched control antibody staining is shown in Figure S6). Scale bars, 10 μm. (D) Immunoblot analysis of IgT, IgM, and IgD on buccal bacteria. Lane 1, 0.1 μg of purified IgT, IgM, or IgD; lanes 2–7, buccal bacteria (n = 6). (E) Percentage of total buccal mucus IgT, IgM, or IgD coating buccal bacteria (n = 12). The median is shown by a red line. Data are representative of at least three independent experiments. ANOVA, analysis of variance.

Previous studies have reported that diverse microbial communities colonize the mucosal surfaces of teleosts, and sIgT is known to coat a large percentage of microbiota on the mucosal surfaces of trout (Xu et al., 2016). To analyze the role of buccal sIgT in recognizing and coating the buccal microbiota, we isolated buccal-associated bacteria and measured their levels of coating by trout sIgM, sIgT, or sIgD. Flow cytometry analysis showed that a large percentage of buccal-associated bacteria were prevalently stained for IgT (∼35%), followed by IgM (∼20%), and to a much lesser extent, IgD (∼10%) (Figures 2A and 2B). Importantly, immunofluorescence microscopy substantiated the results obtained by flow cytometry (Figure 2C; isotype-matched control antibodies, Figure S6). Moreover, immunoblot analysis further confirmed the presence of IgT, IgM, or IgD on these bacteria (Figure 2D). Interestingly, similar to the results previously reported for trout skin microbiota, we found that more than 50% of total IgT present in the buccal mucus was found coating bacteria, whereas only ∼20% of IgM and ∼17% of IgD was being used for bacterial coating (Figure 2E).

### Trout Buccal Infection with Ich Elicits Strong Local Immune Responses

**Figure 3:**
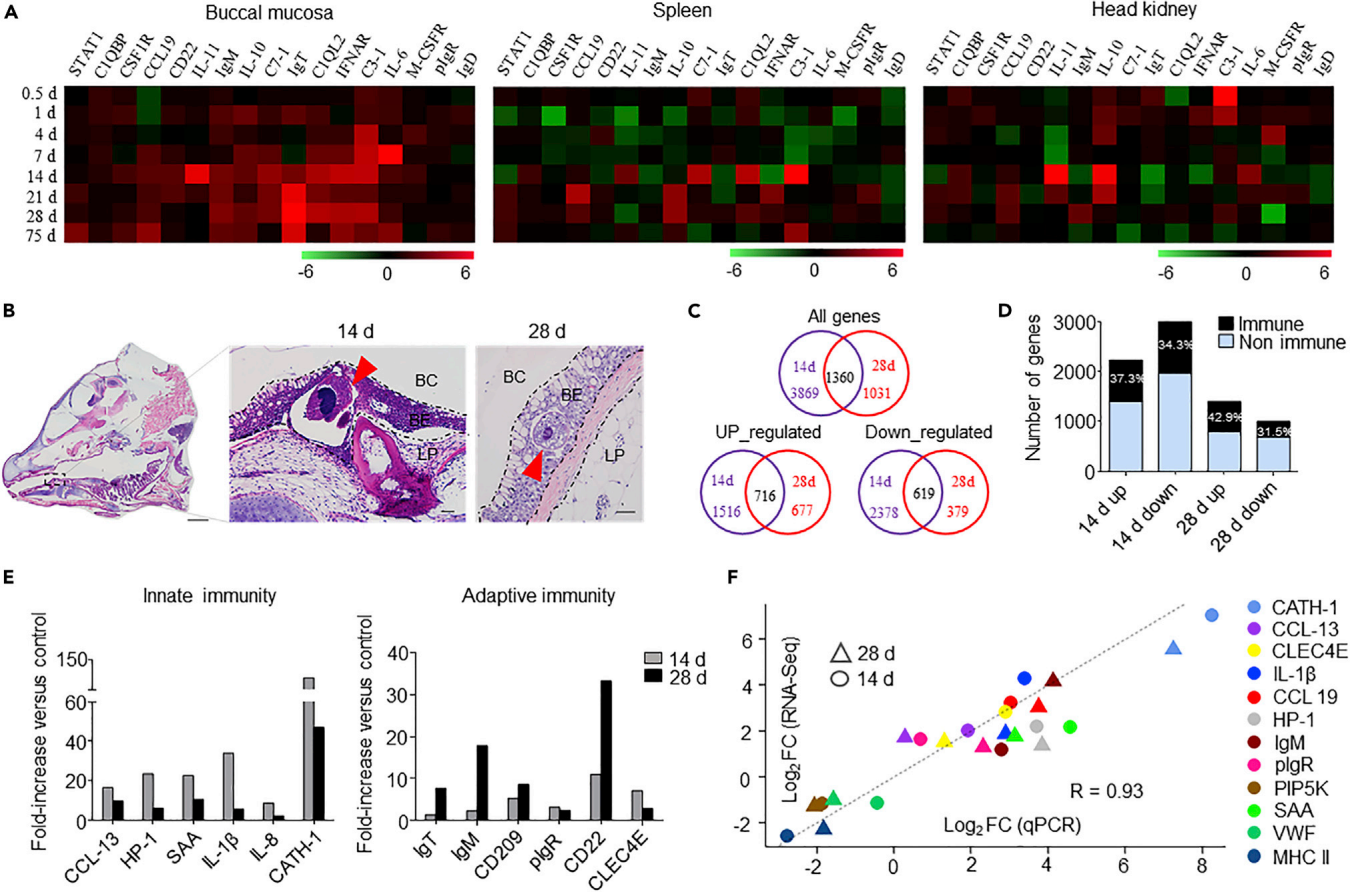
Kinetics of the Immune Response in the BM of Trout Infected with Ich (A) Heatmap illustrates results from quantitative real-time PCR of mRNAs for selected immune markers in Ich-infected fish versus control fish measured at 0.5, 1, 4, 7, 14, 21, 28, and 75 days post infection (n = 6 per group) in the BM (left), spleen (middle), and head kidney (right) of rainbow trout. Data are expressed as mean fold increase in expression. (B) Histology of trout BM at days 14 and 28 post infection with Ich. Red arrows indicate Ich parasite. BC, buccal cavity; BE, buccal epithelium; LP, lamina propria. Scale bars, 3 mm (left), 50 μm (middle and right). (C) Venn diagrams of RNA-seq experiment representing the overlap of genes upregulated or downregulated in the BM of rainbow trout 14 or 28 days after infection with Ich versus control fish. (D) Percentage (mean) of immune and non-immune genes after the differentially expressed genes filtered by rainbow trout immune genes libraries (n = 9 per group). (E) Representative innate and adaptive immune genes modulated by Ich infection at days 14 and 28 post infection (n = 9 per group). Data are expressed as mean fold increase in expression. (F) Confirmation of RNA-seq studies by qPCR of mRNAs of twelve selected genes in the BM of rainbow trout (n = 9 per group). Data are expressed as mean log_2_ (fold change) in expression.

We next evaluated the kinetics of the immune responses that take place in the BM after bath infection with the Ich parasite. By qPCR, we measured the expression of 12 immune-related genes and cell markers in the BM, head kidney, and spleen of trout at 0.5, 1, 4, 7, 14, 21, 28, and 75 days post infection (dpi) (Figure 3A). These studies showed that strong immune responses were generated in not only the head kidney and spleen but also the BM (Figure 3A). Histological examination showed that Ich theronts started appearing on the buccal surface of trout at 14 dpi (Figure 3B). Notably, days 14 and 28 were the most relevant in terms of the intensity of the immune response, and therefore, these two time points were selected for high-throughput transcriptome sequencing of the BM. RNA-sequencing (RNA-seq) libraries made from 12 samples that separately represented four groups (C14d, day 14 control group; C28d, day 28 control group; E14d, day 14 exposed to Ich group; E28d, day 28 exposed to Ich group) were sequenced on an Illumina platform (Bentley et al., 2008). The expression of a total of 5,229 (day 14) and 2,391 (day 28) genes was significantly modified following Ich infection, with 2,232 and 1,393 genes upregulated and 2,997 and 998 genes downregulated at days 14 and 28, respectively (Figure 3C). After filtering by the *Oncorhynchus mykiss* immune gene library, more than 30% of differentially expressed genes (DEGs) were identified as immune-related genes, as shown in the histogram (Figure 3D). To further investigate the DEGs of the BM that were involved in responding to Ich infection among the four groups, KEGG pathway analysis was conducted. Interestingly, we found that pathways associated with immune response, signal molecules, infectious disease, and metabolism were all overrepresented in the differentially expressed set of genes (Tables S2 and S3). Importantly, we identified a significant modification in the expression of genes (Figure S7) involved in innate immunity (Figure 3E, left; Table S4) and adaptive immunity (Figure 3E, right; Table S4) on both days 14 and 28 following Ich infection. Moreover, to validate the DEGs identified by RNA-seq, 12 candidate genes (9 upregulated and 3 downregulated) were selected for qPCR confirmation. As shown in Figure 3F, the qPCR results were significantly correlated with the RNA-seq results at each time point (correlation coefficient 0.93, p < 0.001).

### Response and Proliferation of B cells in Trout BM after Ich Parasite Infection

**Figure 4:**
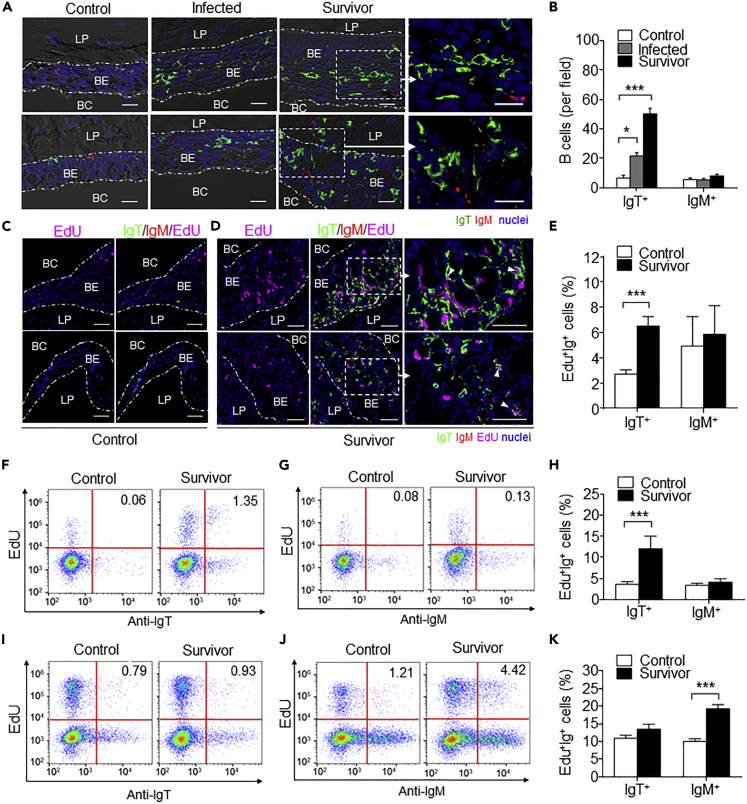
Increases and Proliferative Responses of IgT^+^ B cells in the BM of Trout Infected with Ich (A) Two different DIC images of immunofluorescence staining on paraffinic sections of BM from uninfected control fish (left), 28 days infected fish (middle), survivor fish (right), and enlarged images of the areas outlined, stained for IgT (green) and IgM (red); nuclei are stained with DAPI (blue). BC, buccal cavity; BE, buccal epithelium; LP, lamina propria. Scale bar, 20 μm. Data are representative of at least three different independent experiments (n = 12 per group). (B) IgT^+^ and IgM^+^ B cells in paraffinic sections of BM from uninfected control fish, infected fish, and survivor fish (n = 12), counted in 25 fields (original magnification, ×40). (C and D) Immunofluorescence analysis of EdU incorporation by IgT^+^ or IgM^+^ B cells in the BM of control (C) and survivor fish (D). Paraffinic sections of BM were stained for EdU (magenta), trout IgT (green), trout IgM (red), and nuclei (blue) detection (n = 9 per group). White arrowheads point to cells double stained for EdU and IgT. BC, buccal cavity; BE, buccal epithelium; LP, lamina propria. Scale bars, 20 μm. (E) Percentage of EdU^+^ cells from the total BM IgT^+^ or IgM^+^ B cell populations in control or survivor fish counted from C and D. (F and G) Representative flow cytometry dot plot showing proliferation of IgT^+^ B cells (F) and IgM^+^ B cells (G) in BM leukocytes of control and survivor fish (n = 12 per group). (H) Percentage of EdU^+^ cells from the total BM IgT^+^ or IgM^+^ B cell populations in control or survivor fish (n = 12). (I and J) Representative flow cytometry dot plot showing proliferation of IgT^+^ B cells (I) and IgM^+^ B cells (J) in head kidney leukocytes of control and survivor fish (n = 12 per group). The percentage of lymphocytes representing proliferative B cells (EdU^+^) is shown in each dot plot. (K) Percentage of EdU^+^ cells from the total head kidney IgT^+^ or IgM^+^ B cell populations in control or survivor fish (n = 12 per group). *p < 0.05, ***p < 0.001 (one-way ANOVA with Bonferroni correction). Data in B, E, H, and K are representative of at least three independent experiments (Mean ± SEM).

Using immunofluorescence microscopy, we observed few IgT^+^ and IgM^+^ B cells in the buccal epithelium of control fish (Figure 4A, left; isotype-matched control antibodies, Figure S4B). Interestingly, a moderate increase in the number of IgT^+^ B cells was observed in the buccal epithelium of trout from the infected group (28 dpi) (Figure 4A, middle). Notably, a large number of IgT^+^ B cells accumulated in the buccal epithelium of survivor fish (75 dpi) when compared with those of control fish (Figure 4A, right). Cell counts of the stained sections described in Figure 4A showed that the IgT^+^ B cells increased ∼3-fold and ∼8-fold in the infected and survivor fish, respectively (Figure 4B). However, the abundance of IgM^+^ B cells did not change significantly in the infected and survivor fish when compared with the controls (Figures 4A and 4B).

Next, we investigated whether the increase of IgT^+^ B cells observed in the BM of survivor fish was derived from the process of local IgT^+^ B cell proliferation or due to an infiltration of these cells from systemic lymphoid organs. To do so, we measured the *in vivo* proliferative responses of IgT^+^ and IgM^+^ B cells stained with 5-Ethynyl-2′-deoxyuridine (EdU), a thymidine analogue that incorporates into DNA during cell division (Salic and Mitchison, 2008). By immunofluorescence microscopy analysis, we observed a significant increase in the proliferation of EdU^+^ IgT^+^ B cells in survivor fish (∼6.35 ± 0.34%) when compared with that of the control fish (∼2.62 ± 0.06%) (Figures 4C–4E). However, no difference was detected in the percentage of EdU^+^ IgM^+^ B cells between control fish and survivor fish (Figures 4C–4E). Similarly, by flow cytometry, we found a significant increase in the percentage of EdU^+^ IgT^+^ B cells in the BM of survivor fish (∼12.11 ± 1.32% in total IgT^+^ B cells) when compared with that of control fish (∼4.01 ± 0.34% in total IgT^+^ B cells) (Figures 4F–4H). On the contrary, we did not observe any significant difference in the percentage of EdU^+^ IgM^+^ B cells between control and survivor fish (Figures 4F–4H). Interestingly, a large increase in the percentage of EdU^+^ IgM^+^ B cells in the head kidney of survivor fish were detected when compared with that of control fish, whereas the percentage of EdU^+^ IgT^+^ B cells did not show a significant difference between the two groups (Figures 4I–4K).

### Ig Responses in Trout BM after Ich Parasite Infection

**Figure 5:**
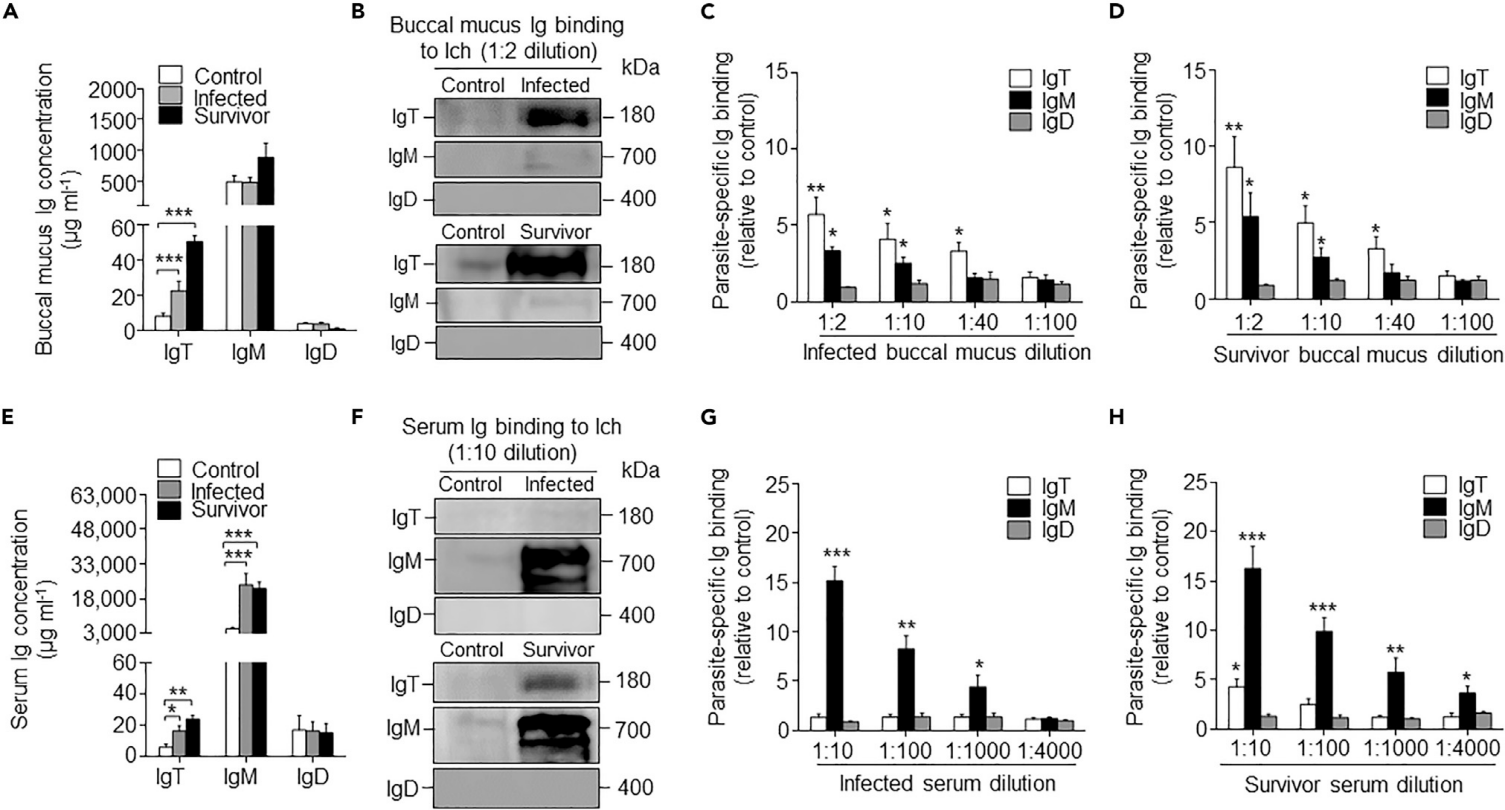
Immunoglobulin Responses in the Buccal Mucus and Serum from Infected and Survivor Fish (A) Concentration of IgT, IgM, and IgD in buccal mucus of control, infected, and survivor fish (n = 12 per group). (B) Immunoblot analysis of IgT-, IgM-, and IgD-specific binding to Ich in buccal mucus (dilution 1:2) from infected and survivor fish. (C and D) IgT-, IgM-, and IgD-specific binding to Ich in dilutions of buccal mucus from infected (C) and survivor (D) fish, evaluated by densitometric analysis of immunoblots and presented as relative values to those of control fish (n = 9 per group). (E) Concentration of IgT, IgM, and IgD in serum of control, infected, and survivor fish (n = 12 per group). (F) Immunoblot analysis of IgT-, IgM-, and IgD-specific binding to Ich in serum (dilution 1:10) from infected and survivor fish (n = 12 per group). (G and H) IgT-, IgM-, and IgD-specific binding to Ich in dilutions of serum from infected (G) and survivor (H) fish, evaluated by densitometric analysis of immunoblots and presented as relative values to those of control fish (n = 9 per group). *p < 0.05, **p < 0.01, and ***p < 0.001 (unpaired Student's t test). Data in A, C, D, E, G, and H are representative of at least three independent experiments (Mean ± SEM).

To investigate whether parasite-specific Igs were produced in trout after Ich parasite challenge, we measured the Igs concentration and the capacity of Igs from buccal mucus and serum to bind to the parasite. Immunoblot analysis showed that the IgT concentration in the buccal mucus from infected and survivor fish increased by ∼2- and ∼8-fold, respectively, when compared with control fish, whereas the concentration of IgM and IgD did not change significantly in any fish groups (Figure 5A). In contrast, only ∼2- and 3-fold increases of serum IgT concentration were observed in infected and survivor fish, respectively, whereas the concentration of serum IgM increased by ∼5-fold in both the infected and survivor groups when compared with control fish (Figure 5E). However, in both infected and survivor fish, the concentration of IgD did not change significantly in either the buccal mucus or serum (Figures 5A and 5E). By a pull-down assay, we found a significant increase in parasite-specific IgT binding in up to 1/40 diluted buccal mucus of infected and survivor fish, in which we detected ∼3.8-fold and ∼3.7-fold binding increases, respectively, when compared with that of the control fish (Figures 5B–5D). Conversely, in serum, parasite-specific IgT binding was detected only in 1/10 dilution of the survivor fish (Figures 5F–5H). In contrast, parasite-specific IgM binding was detected in up to 1/1,000 (∼4.8-fold) and ¼,000 (∼4.2-fold) of the diluted serum from infected and survivor fish, respectively. Finally, in both the infected and survivor fish, we could not detect any parasite-specific IgD binding in the buccal mucus or serum (Figures 5B–5D and 5F–5H).

The substantial increase of proliferating IgT^+^ B cells and high parasite-specific IgT responses occurred in the BM of survivor fish, suggesting that specific IgT might be locally generated in the BM of trout. To further test this hypothesis, we measured the parasite-specific Igs titers from the medium of cultured BM, head kidney, and spleen explants from control and survivor fish (Figure S8). Importantly, we found a significant increase in parasite-specific IgT binding in up to 1/10 diluted medium (∼3.6-fold) of cultured BM explants of survivor fish, whereas parasite-specific IgM binding was observed only at the 1/2 dilution in the same medium (Figures S8A and S8D). In contrast, predominant parasite-specific IgM binding was observed in up to 1/40 dilutions in the medium of head kidney and spleen explants, and parasite-specific IgT binding was detected in up to 1/10 dilutions in the same medium (Figures S8B, S8C, S8E, and S8F). Interestingly, negligible parasite-specific IgD binding was detected in the medium of cultured BM, head kidney, and spleen explants from survivor fish (Figures S8A–S8F).

**Figure 6:**
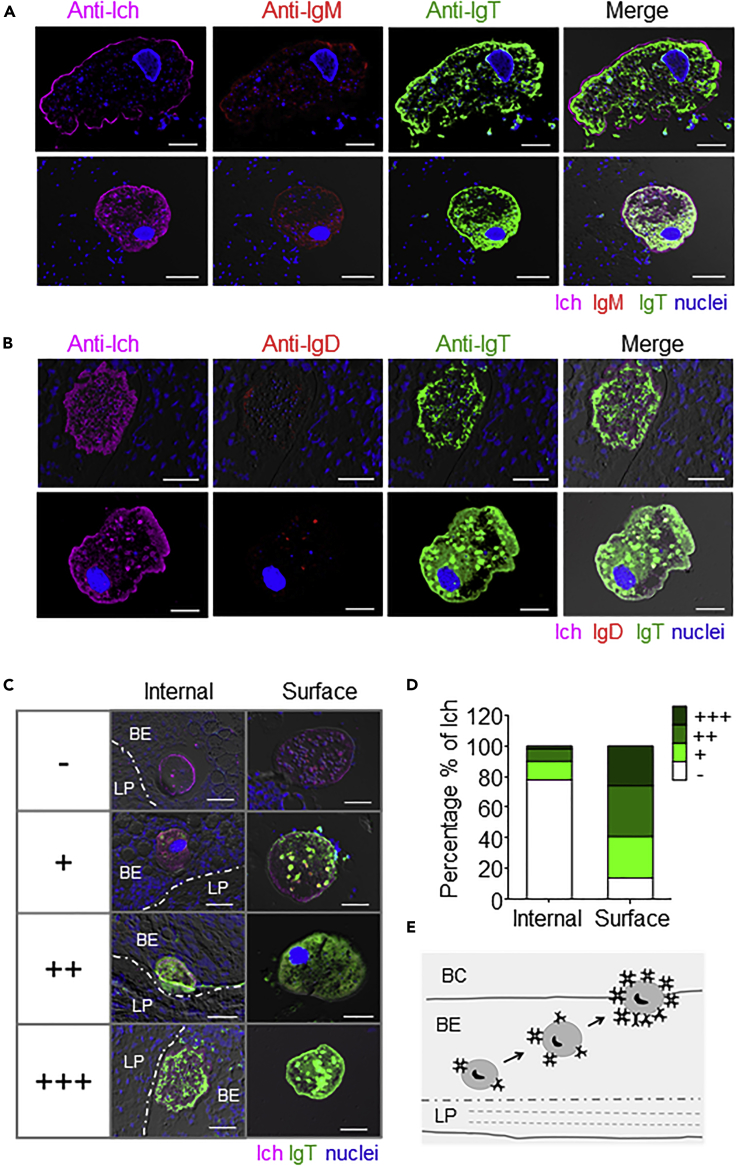
Parasites Are Predominantly Coated by IgT in the Buccal Epithelium of Infected Trout (A and B) Four different microscope images of slides showing immunofluorescence staining of Ich parasites in BM paraffinic-sections from trout after 28 days of infection with Ich (n = 6). From left to right: Ich (magenta), IgM (red), and IgT (green) with nuclei stained with DAPI (blue) (A); From left to right: Ich (magenta), IgD (red), and IgT (green) with nuclei stained with DAPI (blue) (B). DIC images showing merged staining (prebleed control and anti-Ich antibodies are shown in Figure S4C). Scale bars, 50 μm. (C) The different levels of Ich parasites coated by IgT (green) in the inside or surface of BM were divided into four main categories. –, no coating; +, slight level of coating; ++, high level of coating, and +++, strong level of coating. Buccal epithelium (BE) and lamina propria (LP) are shown. Scale bars, 50 μm. (D) Percentage of different levels of Ich parasites coated by IgT in the inside (n = 50) or surface (n = 70) of BM. (E) Proposed model of Ich parasites coated by IgT in the epithelium of BM. Buccal cavity (BC), buccal epithelium (BE), and lamina propria (LP) are shown. Data are representative of at least three different independent experiments.

The high expression of local parasite-specific IgT and large increases in the number of IgT^+^ B cells in the BM of trout after Ich parasite challenge led us to hypothesize a dominant role of IgT in buccal immunity. At 28 dpi, infected fish showed small white dots on the buccal surface, and using immunofluorescence microscopy, Ich trophonts were easily detected in the buccal epithelium of these fish using an anti-Ich antibody (Figures 6A and 6B; prebleed control antibodies, Figure S4C). Interestingly, we found that most parasites in the BM were intensely stained with IgT, whereas only some parasites were slightly recognized by IgM and nearly no parasites were coated with IgD (Figures 6A and 6B). Interestingly, we found that the levels of IgT coating on Ich parasites located inside of the buccal epithelium differed from those on the surface of the buccal epithelium (Figures 6C and 6D). The lower percentage of low (12%), medium (8%), and high levels (2%) of IgT coating on Ich parasites within the buccal epithelium than those (low, 27%; medium, 33%; high, 26%) located on the surface of the buccal epithelium (Figure 6D), respectively, suggests that IgT plays a key role in forcing the Ich parasite to exit from the epithelium of trout (Figure 6E).

### pIgR in Trout BM

In mammals, sIgA can be transepithelially transported by pIgR into the BM (Brandtzaeg, 2013). In trout, we have previously reported that tSC, the secretory component of trout pIgR (tpIgR), is associated with sIgT in the gut, gills, skin, and nose (Zhang et al., 2010, Xu et al., 2013, Xu et al., 2016, Tacchi et al., 2014). Importantly, using immunofluorescence microscopy, most tpIgR-containing cells were observed in the buccal epithelium layer of adult control rainbow trout using anti-tpIgR antibody (Figure 1I). Therefore, we hypothesized that tSC plays a key role in the transport of sIgT into the BM of trout. By immunoblot analysis, we detected tSC in the buccal mucus but not in the serum (Figure S9A). To determine whether buccal mucus sIgT was associated with tSC, using antibodies against tSC (trout pIgR) and IgT, we carried out co-immunoprecipitation assays in buccal mucus from survivor fish. Our results showed that antibodies against trout IgT were able to co-immunoprecipitate tSC in the buccal mucus (Figure S9B). Moreover, sIgT in the buccal mucus could also be immunoprecipitated by anti-pIgR antibody (Figure S9C). Using immunofluorescence microscopy, we observed that most pIgR-containing cells were located in the buccal epithelium of control trout, as shown also in Figure 1I. Critically, some of those pIgR-containing cells were also positively stained with anti-IgT antibody, thus supporting further a role of pIgR in the transport of sIgT into the BM (Figure S9D).

## Discussion

Mucosal immunoglobulins (sIgs), especially sIgA responses in the saliva of the BM of mammals have been extensively reported (Brandtzaeg, 2007, Brandtzaeg, 2013). However, nothing is known with regards to the evolution and roles of sIgs and B cells at the BM of early vertebrates. In this study, we first show that the trout BC contains a MALT characterized by an epithelium layer containing a higher percentage of IgT^+^ B cells than IgM^+^ B cells, similar to what we have previously reported in the fish gut, skin, gills, and nose (Zhang et al., 2010, Xu et al., 2013, Xu et al., 2016, Tacchi et al., 2014). Interestingly, the concentration of buccal mucus sIgT was found to be higher than that reported in the skin, gills, and nose mucus (Xu et al., 2013, Xu et al., 2016, Yu et al., 2018). Therefore, our results indicate that the amount of mucosal Igs differs among the fish mucosal surfaces, similar to the situation of mammalian sIgA, which is found in different concentrations at various mucosal sites (Powell et al., 1977, Okada et al., 1988, Aufricht et al., 1992). Notably, trout IgT was found mainly in polymeric form in the buccal mucus, similar to the finding of sIgA in the saliva from humans (Brandtzaeg, 2013). In contrast, trout sIgD was in monomeric form in the buccal mucus, as previously found in the gills and nose (Xu et al., 2016, Yu et al., 2018). In line with the descriptions of other sources of mucus in teleosts (Xu et al., 2016), all subunits of polymeric sIgT in trout buccal mucus were associated by non-covalent interactions. It is worth pointing out that, in agreement with the finding that the ratio of IgA/IgG in saliva is much higher than that in serum in mammals (Brandtzaeg, 2004), we found that the ratio of IgT/IgM in the buccal mucus was 25-fold higher than in the serum. Thus, the predominance of IgT^+^ B cells and the high IgT/IgM ratio in the trout BM indicate a potential role for sIgT in mucosal buccal immune responses. Moreover, similar to the transport mechanism for sIgA in the BM from mammals through the pIgR (Proctor and Carpenter, 2002), we observed that pIgR-positive cells exist in the epithelial layer of the BM in rainbow trout and that trout pIgR was associated with sIgT in buccal mucus.

In mammals, the BM surface is colonized by high densities of microbiota, suggesting a tight cross talk between the microbiota and the buccal epithelium (Isogai et al., 1985, Beem et al., 1991, Gadbois et al., 1993, Marcotte and Lavoie, 1998). To prevent microbiota translocation from the buccal mucus into the epithelium, sIgA plays a key role in immune exclusion by coating a large fraction of the bacterial microbiota (Marcotte and Lavoie, 1998). Here, we show that sIgT is the main Ig class coating bacteria from buccal microbiota while a significantly lower percentage of the microbiota was coated by both sIgM and sIgD. This result is in agreement with previously reported findings in trout gut, skin, gills, and nose microbiota (Zhang et al., 2010, Xu et al., 2013, Xu et al., 2016, Tacchi et al., 2014). Interestingly, it has been reported that salivary sIgA predominantly coats the surface of bacterial pathogens, such as *Streptococcus mutans*, *Actinobacillus actinomycetemcomitans*, and *Porphyromonas gingivalis*, which are strongly associated with oral diseases in mammals (Marcotte and Lavoie, 1998, Nogueira et al., 2005, Mikuls et al., 2009). Hence, to gain insight into the role of sIgT in the homeostasis of BM, future studies are needed to ascertain the type of buccal microbiota species coated by sIgT.

Here, we show also a key involvement of buccal sIgT in the immune response against Ich, a trout mucosal pathogen. Interestingly, the capacity of Ich to invade the BM of fish had never been appreciated to date. Following Ich infection, the upregulation of both innate and adaptive immune genes was detected in the trout BM, thus showing the involvement of teleost BM in immunity. It is worth noting that we found that B cell markers (i.e., IgT, IgM, CD22) but not T cells markers are significantly upregulated after infection with Ich. This may indicate that B cells but not T cells play a key role against Ich infection. Alternatively, it is possible that T cell responses were absent in the two time points used for transcriptome analysis, although we cannot exclude the possibility that T cells may still be involved in the immune response against Ich. Moreover, we found a large accumulation of IgT^+^ but not IgM^+^ B cells appearing in the buccal epithelium of infected and survivor fish, whereas a few scattered cells could also be observed in the lamina propria. In contrast, sIgA-secreting cells are localized for the most part in the lamina propria of salivary glands in mammals (Deslauriers et al., 1985, Brandtzaeg, 2007, Brandtzaeg, 2013). Importantly, these findings are in agreement with the increased concentration of IgT but not IgM or IgD at the protein level in the buccal mucus of the same individual, thus indicating that large increases in the concentration of IgT were produced by the accumulation of IgT^+^ B cells in the buccal epithelium. Moreover, high parasite-specific IgT titers were detected in buccal mucus, whereas predominant parasite-specific IgM responses were particularly detected in serum. Thus, our findings in the teleost BM reinforce the notion that IgT and IgM responses are specialized in mucosal and systemic areas, respectively (Zhang et al., 2010, Xu et al., 2013, Xu et al., 2016, Yu et al., 2018). However, previous studies showed that IgT is also involved in immune responses in trout spleen upon systemic viral infection (Castro et al., 2013). Interestingly, we found that the parasite-specific IgT titers in buccal mucus were higher than those previously reported in skin mucus (Xu et al., 2013) but lower than those found in gill and nasal mucus (Xu et al., 2016, Yu et al., 2018), suggesting that the degree of the immune response differs depending on the mucosal surfaces. In addition, in this study we found significant proliferative IgT^+^ B cell responses in the BM of trout, similar to what we have previously reported in the fish gill and nose (Xu et al., 2016, Yu et al., 2018). These results suggest that the accumulation of IgT^+^ B cells in these mucosal surfaces after infection is due to local proliferation, although this remains to be fully demonstrated. However, no studies on IgT^+^ B cell local proliferation have been carried out so far in gut and skin mucosal areas. Thus, future studies are needed to investigate whether similar IgT^+^ B cells proliferative responses are locally observed in the skin and gut of trout upon parasite infection. It is clear, however, that important commonalities are observed in the immune responses thus far studied in the gut, skin, gill, nose, and buccal mucosa, all of which are summarized in Figure S10. Thus, our data strongly suggest that the observed parasite-specific IgT responses in the BM were induced locally as we detected significant proliferative responses of IgT^+^ B cells in the BM upon parasite infection, and supernatants from BM explants of survivor fish contained significant parasite-specific IgT titers. Although these data suggest that IgT-specific responses are induced locally in the BM, at this point we cannot exclude the possibility that many of the BM IgT^+^ B cells have not proliferated locally and that have instead been transferred through blood circulation into the BM after proliferating elsewhere. Further studies are warranted to analyze this important point. In line with what we found in the gill and nose MALTs (Xu et al., 2016, Yu et al., 2018), our data suggest that the trout BM would act both as inductive and effector site of IgT responses. In contrast, mammalian BC appears to act only as an effector site (Jackson et al., 1981, Brandtzaeg, 2007, Novak et al., 2008). In that regard, sIgA is the predominant Ig isotype in human saliva (Brandtzaeg, 2007, Brandtzaeg, 2013), and a dramatic increase of IgA secretion as well as IgA-positive cells in the salivary gland occurs following infection with pathogens, including the HIV virus (Lu and Jacobson, 2007), the bacteria *S. mutans* (Colombo et al., 2016), and the parasite *toxoplasma gondii* (Loyola et al., 2010). Thus, from an evolutionary viewpoint, our findings indicate a conserved role of mucosal Igs (i.e., IgT, IgA) in the control of pathogens at the BM in aquatic and terrestrial vertebrates.

Here we found a larger ratio of high-intensity IgT coating on the Ich parasites located on the surface of BM when compared with that of Ich inside the buccal epithelium. Thus, it is conceivable that the strong parasite-specific IgT responses elicited in the BM after infection force the BM parasites inside the epithelium to exit it (Wang and Dickerson, 2002). In line with this hypothesis, previous studies have demonstrated that passive immunization of channel catfish (*Ictalurus punctatus*) using mouse monoclonal antibodies specific to Ich immobilization antigens contributes to the parasite clearance or exit from the host (Clark et al., 1996, Clark and Dickerson, 1997). However, we cannot exclude the possibility that rather than occurring inside the BM epithelium, the high coating of the parasite by specific IgT occurs outside of the BM epithelium, by parasite-specific IgT present in the BM mucus (i.e., specific IgT would be generated upon infection of fish by the parasite). If coating occurs via IgT present in the mucus outside the BM epithelium, this IgT could then be involved in the immobilization of the parasite, thus preventing it from invading the epithelium. Moreover, we cannot exclude that factors other than IgT may force the parasite to exit the BM epithelium. For example, complement might be activated by IgT bound to the parasite, which in turn might elicit the exit response, or contribute to such response. Future studies are needed to investigate the specific role of IgT coating as well as other immune factors in forcing the exit of Ich from the BM epithelium, or in preventing its invasion.

**Figure 7:**
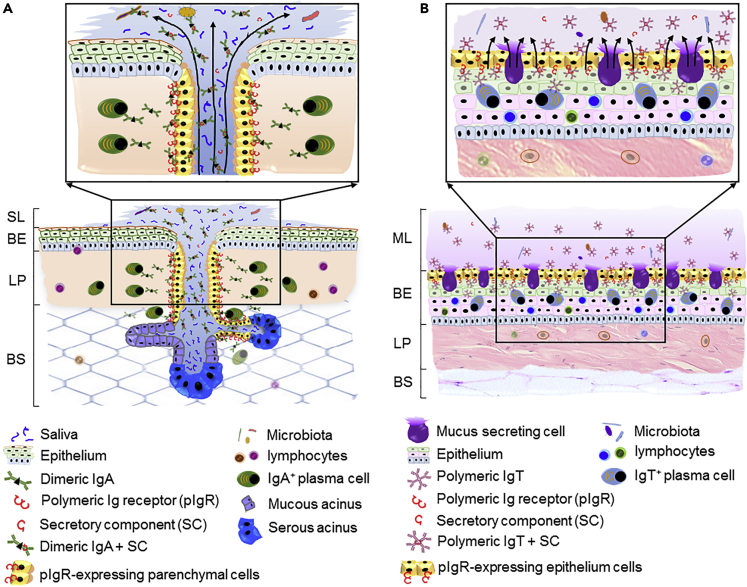
Simplified Scheme of the Analogous Strategies of Mammals and Fish in the Production and Secretion of Immunoglobulin-containing Fluids in their BM (A, lower) In mammals, the BM contains numerous salivary glands, which produce and secrete saliva into the salivary layer (SL) via secretory ducts. Localized aggregations of IgA+ plasma cells are commonly found in the lamina propria (LP) of the BM. (A, upper) Mucosal immunoglobulin (sIgA) containing the joining (J) chain is produced by local IgA^+^ plasma cells in the LP and transported inside salivary gland via pIgR also termed as (membrane secretory component [mSC]) expressed basolaterally on parenchymal cells. Thereafter, sIgA mixes with saliva in the salivary gland and the IgA-containing saliva is secreted into the SL through ductal system. (B, lower) Teleost BM is instead populated with abundant mucus-secreting cells, which produce mucus, which is secreted directly into the mucous layer (ML). IgT-secreting cells are found scattered mainly in the buccal epithelium (BE) where they increase in significant numbers upon infection. (B, upper) Mucosal IgT (sIgT) is secreted by intraepithelial IgT-secreting cells and transported via pIgR-expressing epithelial cells directly into the ML where it mixes with mucus derived from mucus-secreting cells. Finally, the sIgT-containing mucus and sIgA-containing saliva from fish and mammals, respectively, preserve BC homeostasis by maintaining the establishment of a healthy microbiota and at the same time, by fighting potential pathogens.

In conclusion, our findings show the presence of a previously unrecognized bona fide MALT in the BM of a non-tetrapod species and its involvement in both the control of pathogens and recognition of microbiota. Significantly, these data indicate that mucosal adaptive immune responses evolved both in tetrapod and non-tetrapod species through a process of convergent evolution, as fish IgT and mucus-producing cells are evolutionary unrelated to mammalian IgA and salivary glands, respectively. It is in this aspect that fish and mammals have evolved different fascinating strategies in the way by which their immunoglobulin-containing fluids are produced and secreted in the BM (Figure 7). On the one hand, mammalian sIgA produced by lamina propria-plasma cells is transported inside the salivary glands via pIgR-expressing parenchymal cells; the sIgA-containing saliva within the salivary gland is thereafter secreted into the outer layer of the BM epithelium (Figure 7A). In contrast, fish sIgT produced by intraepithelial IgT^+^ B cells is transported via pIgR-expressing epithelial cells into the outer layer of the buccal epithelium where it mixes with mucus derived from mucus-secreting cells (Figure 7B). Thus, different molecules (sIgT versus sIgA) and cell types/glands (mucus-secreting cells versus salivary glands) of fish and mammals utilize different but functionally analogous strategies to coat the outer layer of the BM epithelium with different sIg-containing fluids (mucus versus saliva) with the same goal (the control of pathogens and microbiota). Interestingly, and based on our data, it would appear that the main role of mucus-based buccal fluids in lower vertebrates is immune defense and mucosal homeostasis, whereas throughout evolutionary time, the saliva-based buccal fluids of tetrapods have gained an important role in digestion. Future work is required to further address this attractive. Finally, since we find that sIgT responses are locally produced in the fish BM, from a practical level our findings may have important implications for the design of future fish vaccines that stimulate mucosal BM responses.

### Limitations of the Study

This study shows that a well-defined diffuse MALT is present in the trout's BC, which can produce strong innate and adaptive immune responses to the parasitic infection. Moreover, we provide evidence that specific IgT is the main player involved in the buccal adaptive immunity. However, there are limitations to our study due to some experimental constraints. For instance, even though the upregulated expression of B cell and T cell makers in teleost BM reveal that both of them are involved in the buccal immunity against Ich, we did not address the interaction between B cells and CD4-T cells in teleost BM during pathogenic infection, because of the lack of anti-trout CD4 mAb. In addition, this study shows local proliferative IgT^+^ B cell responses and pathogen-specific IgT production in the BM of a fish species, but we cannot rule out the possibility that, on antigen uptake, loaded BM APCs may migrate into central secondary lymphoid organs (that is, spleen or head kidney) where the resulting activated IgT^+^ B cells may then home into the BM. Thus, further experiments will be required to address those aspects conclusively.

## Methods

All methods can be found in the accompanying Transparent Methods supplemental file.

## Supplemental Information

Document S1. Transparent Methods, Figures S1–S10, and Tables S1–S3

Table S4. List of Selected mRNAs, Grouped According to Functional Classes (Shown in Bold), Found to be Up- and Down-regulated by Buccal Infection with Ich (14 d and 28 d), Related to Figure 3
