# Supplementary material for: Convergent Evolution of Mucosal Immune Responses at the Buccal Cavity of Teleost Fish
Source: iScience. 2019 Aug 24;19:821–35. doi: 10.1016/j.isci.2019.08.034 (PMC6734174; doi:10.1016/j.isci.2019.08.034)
Supplement: Document S1. Transparent Methods, Figures S1–S10, and Tables S1–S3 [file mmc1.pdf]

ISCI, Volume *19*

## Supplemental Information

# Convergent Evolution of Mucosal Immune Responses at the Buccal Cavity of Teleost Fish

**Yong-Yao Yu, Wei-Guang Kong, Hao-Yue Xu, Zhen-Yu Huang, Xiao-Ting Zhang, Li-Guo Ding, Shuai Dong, Guang-Mei Yin, Fen Dong, Wei Yu, Jia-Feng Cao, Kai-Feng Meng, Xia Liu, Yu Fu, Xue-zhen Zhang, Yong-an Zhang, J. Oriol Sunyer, and Zhen Xu**

# Supplemental Information

# Convergent evolution of the mucosal immune response in the buccal mucosa of teleost fish

Yong-Yao Yu, Wei-Guang Kong, Hao-Yue Xu, Zhen-Yu Huang, Xiao-Ting Zhang, Li-Guo Ding, Shuai Dong, Guang-Mei Yin, Fen Dong, Wei Yu, Jia-Feng Cao, Kai-Feng Meng, Xia Liu, Yu Fu, Xue-zhen Zhang, Yong-an Zhang, J. Oriol Sunyer, and Zhen Xu

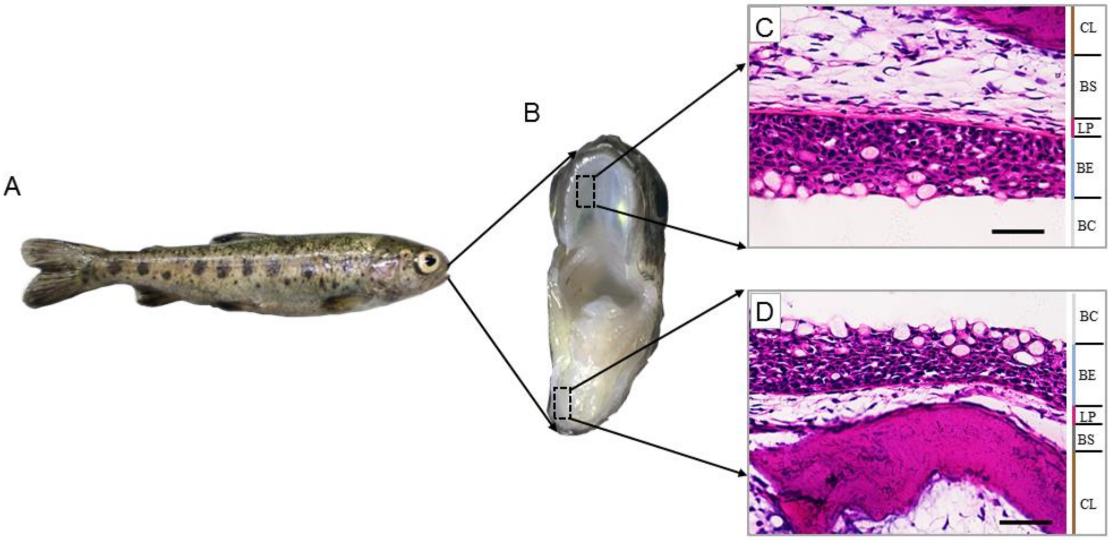


**Figure S1. An overview of trout BM. Related to Figure 1**

Anatomy of rainbow trout (A), BM (B), and paraffin sections of buccal upper mucosa (C) and lower mucosa (D), stained with Haematoxylin/eosin. The black dotted boxes represent the sampling site for paraffin sections. BC, buccal cavity; BE, buccal epithelium; LP, lamina propria; BS, buccal submucosa; CL, cartilage layer. **Scale bar, 50 µm.**

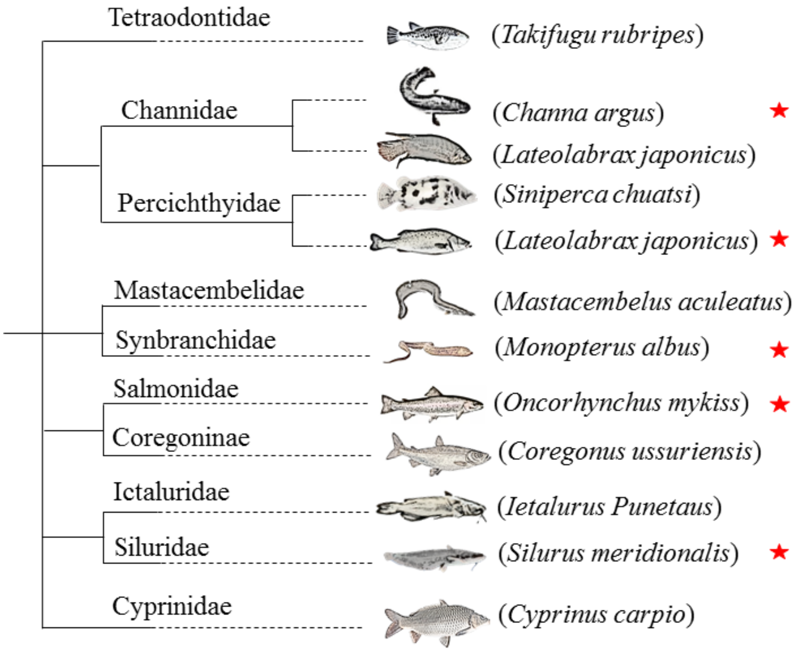


**Figure S2. Candidate fish species from five different families were selected to understand the general organization of teleost BM. Related to Figure 1**

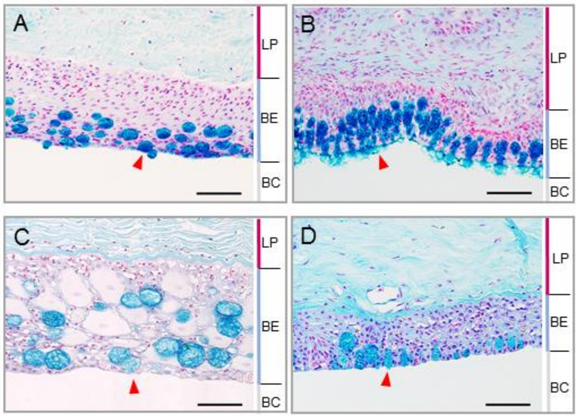


**Figure S3. Abundant mucous cells in teleost BM. Related to Figure 1**

AB staining of the BM of a control adult Japanese seabass (*L. japonicus*) (A), Asian swamp eel (*M. albus*) (B), Southern catfish (*S. meridionalis*) (C), and Snakehead (*C. argus*) (D). Red triangles indicate mucus cells. Scale bar, 50 μm. BC, buccal cavity; BE, buccal epithelium; LP, lamina propria; BS, buccal submucosa.

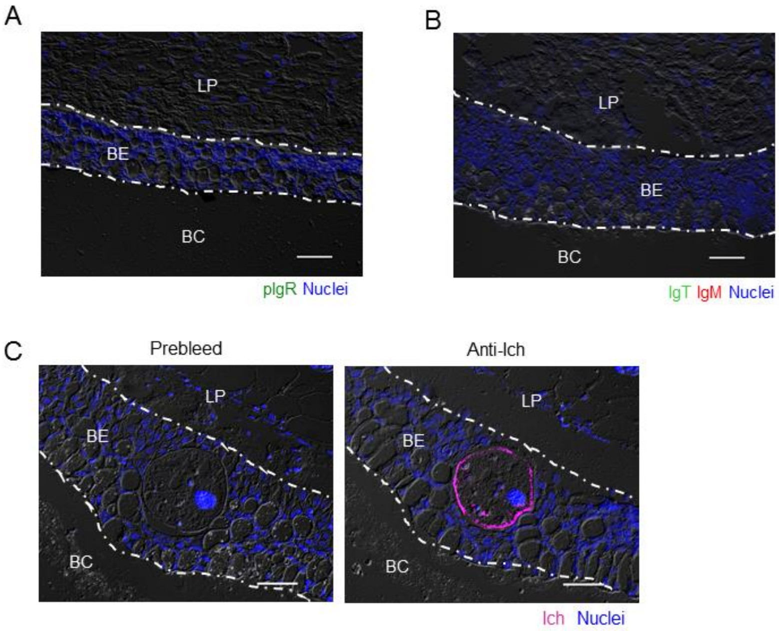


**Figure S4. Isotype control staining for anti-IgT, anti-IgM, anti-pIgR and anti-Ich antibodies in trout BM paraffin-sections. Related to Figure 1**

Differential interference contrast images of buccal paraffin-sections from control fish (A and B) and infected fish, with merged staining of isotype control antibodies for anti-trout pIgR pAb (green, A); or anti-trout IgT pAb (green) and anti-trout IgM (red, B) mAb; or anti-trout Ich pAb (magenta, C). Nuclei were stained with DAPI (blue, A-C). BC, buccal cavity; BE, buccal epithelium; LP, lamina propria. Scale bars, 50 μm. Data are representative of three independent experiments.

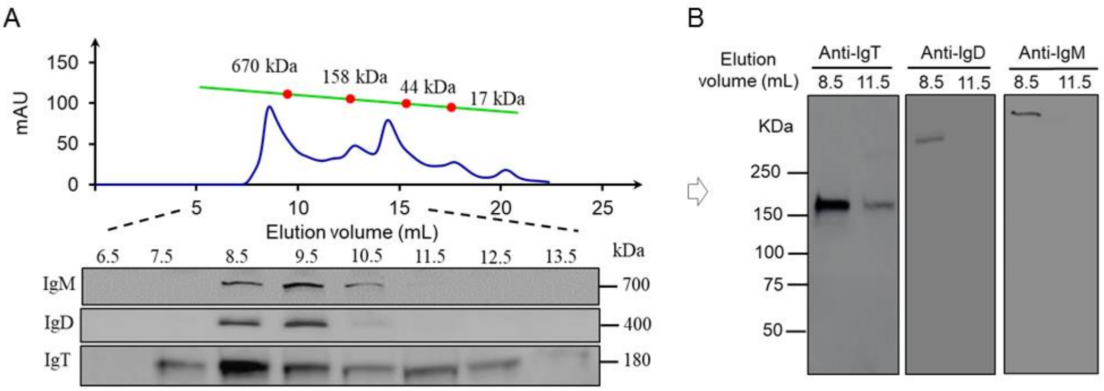


**Figure S5. Protein characterization of buccal mucus immunoglobulins. Related to Figure 1**

(A) Fractionation of buccal mucus (~0.5 mL) by gel filtration (upper) followed by immunoblot analysis of the fractions with anti-trout IgM, anti-trout IgD-specific mAbs or anti-trout IgT-specific pAbs (lower). (B) SDS–PAGE of gel-filtration fractions (4–15%) corresponding to elution volumes of 8.5 and 11.5 mL under non-reducing conditions followed by immunoblot analysis with anti-trout IgM-, anti-trout IgD-specific mAbs or anti-trout IgT-specific pAbs.

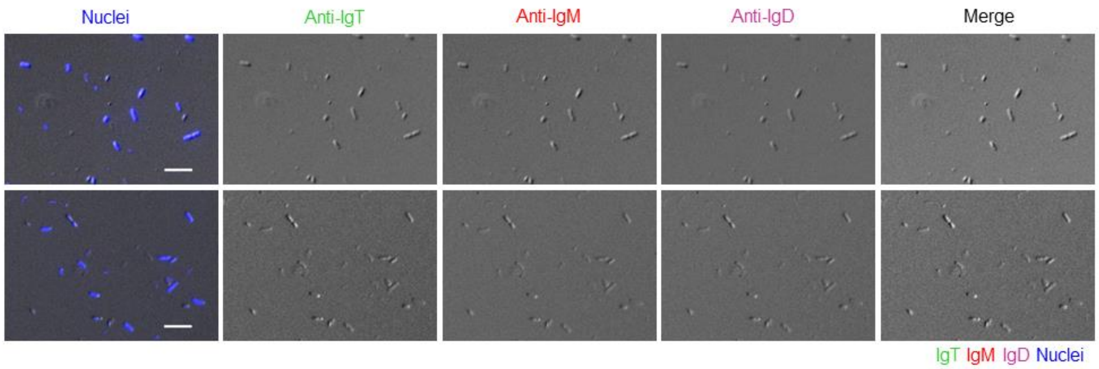


**Figure S6. Staining of trout buccal bacteria with isotype control antibodies for anti-IgT, anti-IgM and anti-IgD mAbs. Related to Figure 2**

Differential interference contrast images (DIC) of buccal bacteria stained with a DAPI-Hoeschst solution (blue), isotype control antibodies for anti-trout IgT (green), for anti-trout IgM (red), or for anti-trout IgD (magenta) mAbs, and merging isotype control antibodies for IgT, IgM and IgD staining. Scale bar, 10 μm. Upper and lower panels display two different samples, representative of at least three independent experiments.

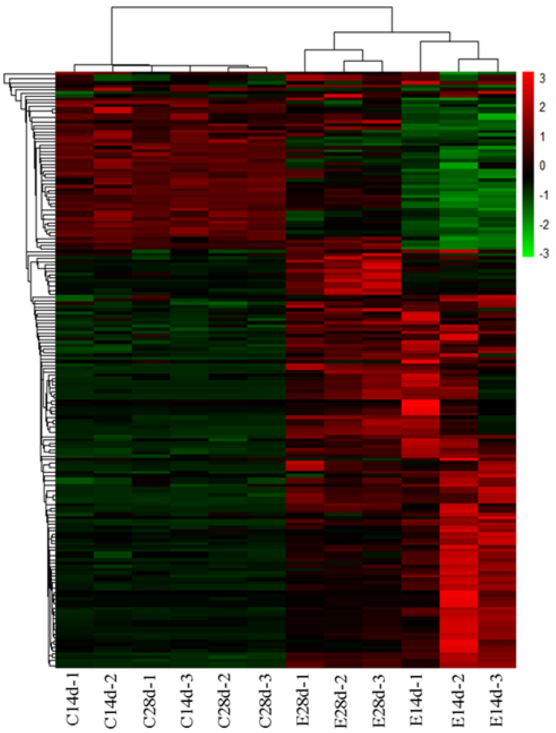


**Figure S7. A heatmap with clustering using the RPKM (Reads Per Kilobase per Million mapped reads) of genes present in Table S4 (C14d, day 14 control group; C28d, day 28 control group; E14d, day 14 exposed to Ich group; E28d, day 28 exposed to Ich group). Related to Figure 3**

Pheatmap package of R (version 3.4.4) was used to picture the heat map, and 'single' method was used to cluster values. The values were scaled in the row direction.

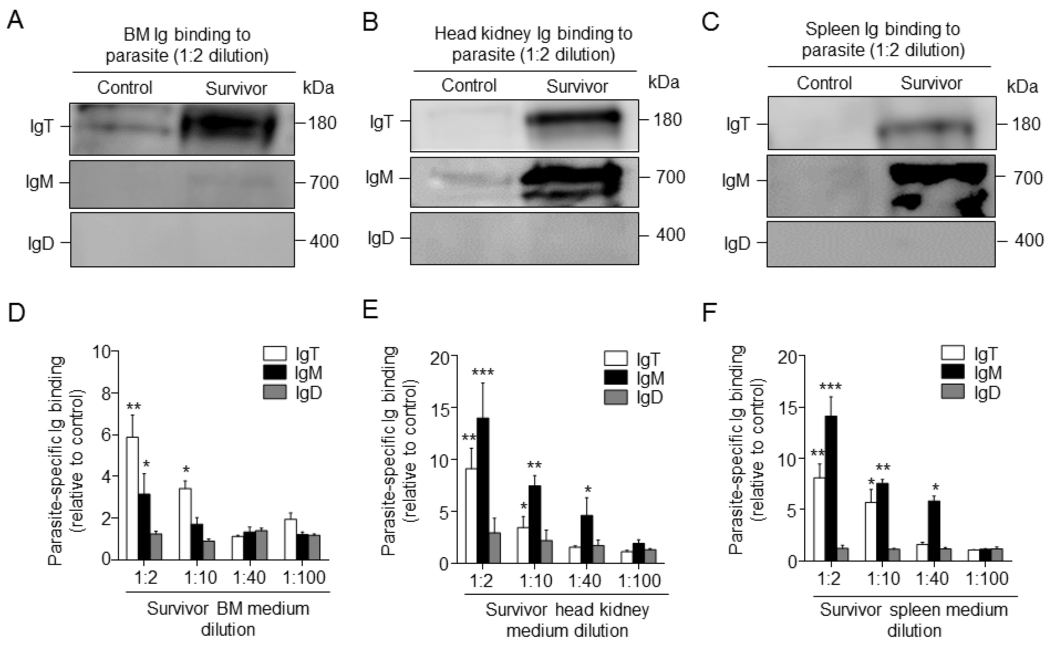


**Figure S8. Local IgT-, IgM- and IgD- specific specific responses in BM explants of survivor fish. Related to Figure 5**

The BM, head kidney, and spleen explants (~25 mg each) from control and survivor fish were cultured for 7 days. Immunoblot analysis of IgT-, IgM- and IgD- specific binding to Ich in the culture medium of BM (A), head kidney (B) and spleen (C) (dilution 1:2) from control and survivor fish. (D-F) IgT-, IgM- and IgD-specific binding to Ich in dilutions of culture medium from BM (D), head kidney (E) and spleen (F) from control and survivor fish, measured by densitometric analysis of immunoblots and presented as relative values to those of control fish (n = 9 per group). *$p < 0.05$, **$p < 0.01$ and ***$p < 0.001$ (unpaired Student's *t*-test). Data are representative of at least three independent experiments (Mean ± SEM).

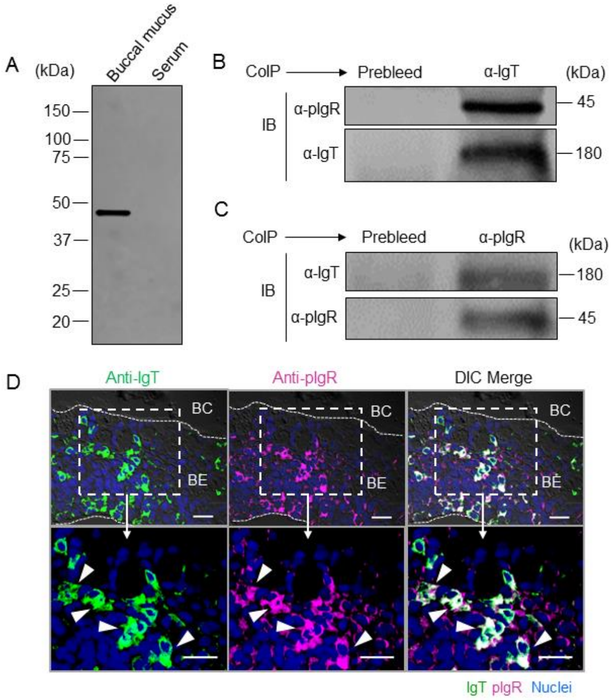


**Figure S9. Trout pIgR associates with buccal sIgT. Related to Figure 1**

(A) SDS-PAGE under reducing conditions of trout serum and buccal mucus, followed by immunoblot analysis using anti-trout pIgR antibody. (B) Co-immunoprecipitation (CoIP) of buccal mucus with anti-trout IgT antibody, followed by immunoblot analysis (IB) under reducing conditions (pIgR detection, upper) or non-reducing conditions (IgT detection, lower). (C) CoIP of buccal mucus with rabbit anti-trout pIgR followed by IB under non-reducing conditions (IgT detection, upper) and reducing conditions (pIgR detection, Lower). IgG purified from rabbit's serum before immunization (Prebleed) served as negative control for rabbit anti-trout pIgR and rabbit anti- trout IgT, respectively (left lane on each panel for B and C). (D) Immunofluorescence staining for pIgR with IgT in BM paraffin-sections of survivor fish. Differential interference contrast images of BM paraffin-sections were stained with anti-trout IgT (green,) anti-trout pIgR (magenta) and DAPI for nuclei (blue) (n = 6). (isotype-matched control antibodies for anti-pIgR in Figure S4A). (E) Enlarged sections of the areas outlined in D without DIC showing some pIgR/IgT colocalization (white triangles). Scale bars, 20 μm. BC, buccal cavity; BE, buccal epithelium. Data are representative of at least three independent experiments.

| | Polymeric IgT | Monomeric IgT | IgT specific titers | IgM specific titers | Accumulation of IgT$^+$ B cell | Accumulation of IgM$^+$ B cell | Proliferation of IgT$^+$ B cell | Proliferation of IgM$^+$ B cell | Microbiota coated with IgT | Microbiota coated with IgM | Microbiota coated with IgD | The transport of IgT is associated to pIgR |
|---|---|---|---|---|---|---|---|---|---|---|---|---|
| Gut M. | +++ | + | ++++ | + | +++ | – | ? | ? | ++++ | ++ | ? | +++ |
| Skin M. | +++ | ++ | ++ | – | +++ | – | ? | ? | +++ | + | ? | +++ |
| Gill M. | +++ | ++ | ++++ | + | +++ | – | ++ | – | ++++ | +++ | + | +++ |
| Nose M. | +++ | ++ | ++++ | + | +++ | – | ++ | – | ++ | ++ | ? | +++ |
| Buccal M. | +++ | ++ | +++ | + | +++ | – | ++ | – | +++ | ++ | + | +++ |
| Serum/Head kidney | – | ++++ | + | +++++ | – | ++ | – | ++ | – | – | – | – |

**Figure S10. Comparative analysis immune responses in different mucosa and systemic organs of teleost fish. Related to Figure 5**

+, low level; ++, medium level; +++, high level; ++++, strong level; -, no detection; ?, unknown, M., mucosa

**Table S1. Primers used in this study. Related to Figures 3**

| Gene | GenBank accession no. | Primer Sequence (5’-> 3’) | |
|---|---|---|---|
| | | **Forward primer** | **Reverse primer** |
| MHCII | DQ246664.1 | GGTGAGTTTGTTGGATAC | AGCGTTAGGCTTACATAGA |
| CCL19 | XM_021602563.1 | GCTGCCACTGTGTTTGTC | CTGTCCTTTCCCTTATGC |
| IgD | JN173049.1 | CAGGAGGAAAGTTCGGCATCA | CCTCAAGGAGCTCTGGTTTGGA |
| pIgR | FJ940682.1 | AGAAGCGTTGGTGTCGTA | AAGCCTTGGTCAGGTCAT |
| IgM | OMU04616 | AAGAAAGCCTACAAGAGGGAGA | CGTCAACAAGCCAAGCCACTA |
| MPO | GBTD01119227 | GCAGAGTCACCAATGACACCA | ATCCACACGGGCATCACCTG |
| FcεRIγ | ACI69533.1 | TACTCCAACTCTCCATCTACTC | CTGTGGATACCCGCCAGTGA |
| GCSFR | AJ616901 | TCCACGGGACAGAGTACCACA | GAAACTGCTTCGATGGCTTCC |
| CD4-1 | AY973028 | TGGTCGAGAGACGATAGATCC | GAGGTACTTGTTTGTGGCATGA |
| MPEG1 | GBTD01065710 | CTCAGACGTGTCCTTCCTCTC | CGTGTATAAGAAGTTACGCACTTG |
| CD4-2b | AY89932 | AAGCCCCTCTTGCCGAGGAA | CTCAACGCCTTTGGTACAGTGA |
| CD3γ | GBTD01057626 | GAACACTGGAATACAAGGACGAGAACAC | GAGCCCCATTTTGCTAGATGTTTTCTT |
| Lck2 | AY973033 | CCTGTTGAAGAGCATTATATTAG | ACGGTTTAGCCGACTGGGTG |
| CD8a | AF178053 | ACTGCCAAGTCGTGCAAAGTG | AAGCCACAGCCAGCAGTCAA |
| TCRa | OMU50991 | CAGCTTGAAGTCAAGAAATAC | TATCAGCACGTTGAAAACGAT |
| MCSFRa | AB091826 | ATCTCCACTCATGGCGACACA | CATCGCACTGGGTTTCTGGTA |
| RORγ1 | NM_001199827.1 | ACAGACCTTCAAAGCTCTTGGTTGTG | GGGAAGCTTGGACACCATCTTTG |
| CD4-2a | AY772711 | CGTGAGAAGTTTGTTGCCGAA | TGGCTGCCTTTGGTACAGTGA |
| Gata3 | NM_001195792.1 | CCAAAAACAAGGTCATGTTCAGAAGG | TGGTGAGAGGTCGGTTGATATTGTG |
| CD8b | AY563420 | TCCTGTATGCTCCAGAACCAG | ATGTTGGGCGAGTTTCTCCG |
| lag3 | XM_021590439.1 | GAGCGTGACATACCACCTACA | ATCAGCTTGCGCCTCCGATA |
| Lck1 | AY973032 | TTCATGGAGAACGGCGCTCT | AGGTCCCGATGGATGTAGTTCTGTTT |
| CD40L | NM_001124666.1 | CAAGCAACCTGTCGTTGGTG | GTACACACGTCTGTCCGGTT |
| IL-6 | CCV01624.1 | ATTTCATCGTTCTCACAGC | ACTACCTCAGCAACCTTCA |
| M-CSFR | NM_001124739.1 | CCCGCCTGTCACCCAATCT | CGTCCCACCAATGCTTCT |
| C3-1 | L24433 | GAGATGGCCTCCAAGAAGATAGAA | ACCGCATGTACGCATCATCA |
| IFNAR | AGO14285.1 | CAGAGCCTCAGGAAGAACT | CAAGGGGTAGAAGAGCATA |
| C1QL2 | XM_021624859.1 | GTCTACTCAAACATCGGC | CATTCTTGGTCAAACACAC |
| IgT | AY870264 | CAGACAACAGCACCTCACCTA | GAGTCAATAAGAAGACACAACGA |
| CD22 | XM_021625667.1 | TGAAGATGACAGTGGCAGAT | GGAGGGTTACAGGTGGAG |
| C7-1 | NM_001124618.1 | TATCTTCACTGCCACGGTC | TAGCCTGTAACTCCACATAGAC |
| IL-10 | NM_001245099.1 | CACCGCCTTCTCCACCATC | CCATAGCGTGACACCCCAC |
| IL-11 | NM_001124382.1 | CAGAGCGTCAAGGAAACAC | GCTCCTGGGAAGACTGTAA |
| CSF1R | NP_001268281.1 | GTGAAGGAGGGCAGTGAT | GATGGTGGCAAACGCAAG |

| | | | |
|---|---|---|---|
| STAT1 | NP_001118179.1 | GACCAGCGAACCCAAGAACCTGAA | CACAAAGCCCAGGATGCAACCAT |
| C1QBP | XM_021617398.1 | CCGCAGTCCGAATTTCTA | GCTTTGTCTCCTTCCGTAT |
| EF-1a | NM_001124339.1 | CAACGATATCCGTCGTGGCA | ACAGCGAAACGACCAAGAGG |
| CATH-1* | NM_001124480.1 | CTGGAGGCAAGCAACAAC | CCCCCAAGACGAGAGACA |
| CCL-19* | KF683302.1 | GTTTCCCTCGCCACTTCAA | GCCACCCACTTGCTCTTTG |
| IL-1β* | NM_001124347.2 | TGATGAATGAGGCTATGGA | GATGGTGAAGGTGGTAAGG |
| CLCE4E* | XM_021562202.1 | GCAGCCACCTTACCATC | CACCCATCTCCAATCCC |
| PIP5K* | XM_021585241.1 | TCCATCGGCCTGGCTTCTAT | TCCTCCTCACGCACCTCCTC |
| VWF* | XM_021580906.1 | AGTGATGAAGGGTGTTGAGG | GTTGCTGCTTAGAAGGTCGT |
| HP-1* | XM_021595153.1 | CGGAGGAGGTTGGAAGC | GCAGCAGAAGCCACAGC |
| CCL-13* | XR_002472294.1 | CAGAACAACCTCCAGTAGC | ATCGTCGTCTTGGCAGTA |
| SAA* | XM_021607573.1 | TTGTTCTGACCCTCGTTG | CCTGGCAGCATCATAGTT |
| IgM* | EF467980.1 | GCTATGGGATGAACTGG | TACCCTGAAATGACTGG |
| MHC II* | XM_021556605.1 | AATGGCGACTGGCACTA | GCCCGATGGCTATCTTA |
| pIgR* | XM_021599266.1 | TGTTACACTCCGCATTCTC | CAGGGCAGGTTTCTGATTT |

*Indicates the isoform specific primers used for validating the differentially expressed genes identified by RNA-Seq.

**Table S2. Pathways involving in response to Ich (14 d). Related to Figure 3**

| KEGG pathway | Description | Input number | Background number | q-value |
|---|---|---|---|---|
| ko04974 | Protein digestion and absorption | 82 | 228 | 3.19E-19 |
| ko04512 | ECM-receptor interaction | 52 | 179 | 4.39E-08 |
| ko04060 | Cytokine-cytokine receptor interaction | 101 | 469 | 8.00E-08 |
| ko00330 | Arginine and proline metabolism | 34 | 102 | 5.74E-07 |
| ko04510 | Focal adhesion | 87 | 466 | 4.33E-04 |
| ko05144 | Malaria | 26 | 91 | 5.53E-04 |
| ko00680 | Methane metabolism | 17 | 48 | 6.40E-04 |
| ko04640 | Hematopoietic cell lineage | 33 | 134 | 7.49E-04 |
| ko05217 | Basal cell carcinoma | 34 | 149 | 2.49E-03 |
| ko05414 | Dilated cardiomyopathy (DCM) | 44 | 213 | 2.96E-03 |
| ko00010 | Glycolysis / Gluconeogenesis | 27 | 116 | 7.12E-03 |
| ko04360 | Axon guidance | 78 | 459 | 9.20E-03 |
| ko04151 | PI3K-Akt signaling pathway | 123 | 802 | 1.92E-02 |
| ko05020 | Prion diseases | 16 | 60 | 2.02E-02 |
| ko04978 | Mineral absorption | 22 | 97 | 2.74E-02 |
| ko00220 | Arginine biosynthesis | 13 | 46 | 2.87E-02 |
| ko05146 | Amoebiasis | 36 | 188 | 2.87E-02 |
| ko00051 | Fructose and mannose metabolism | 17 | 69 | 2.89E-02 |
| ko04514 | Cell adhesion molecules (CAMs) | 58 | 345 | 3.59E-02 |
| ko05205 | Proteoglycans in cancer | 78 | 489 | 3.59E-02 |
| ko04657 | IL-17 signaling pathway | 33 | 175 | 4.33E-02 |
| ko04260 | Cardiac muscle contraction | 35 | 189 | 4.39E-02 |

**Table S3. Pathways involving in response to Ich (28 d). Related to Figure 3**

| KEGG pathway | Description | Input number | Background number | q-value |
|---|---|---|---|---|
| ko00010 | Glycolysis / Gluconeogenesis | 23 | 116 | 1.80E-06 |
| ko04610 | Complement and coagulation cascades | 23 | 131 | 9.77E-06 |
| ko04514 | Cell adhesion molecules (CAMs) | 41 | 345 | 1.01E-05 |
| ko04640 | Hematopoietic cell lineage | 22 | 134 | 3.12E-05 |
| ko05150 | Staphylococcus aureus infection | 14 | 61 | 4.88E-05 |
| ko05414 | Dilated cardiomyopathy (DCM) | 28 | 213 | 6.89E-05 |
| ko04260 | Cardiac muscle contraction | 25 | 189 | 1.64E-04 |
| ko05410 | Hypertrophic cardiomyopathy (HCM) | 26 | 201 | 1.64E-04 |
| ko04512 | ECM-receptor interaction | 24 | 179 | 1.76E-04 |
| ko00680 | Methane metabolism | 11 | 48 | 3.59E-04 |
| ko04261 | Adrenergic signaling in cardiomyocytes | 35 | 366 | 2.49E-03 |
| ko04530 | Tight junction | 39 | 441 | 4.95E-03 |
| ko05143 | African trypanosomiasis | 12 | 77 | 6.10E-03 |
| ko04971 | Gastric acid secretion | 20 | 178 | 7.48E-03 |
| ko04970 | Salivary secretion | 19 | 177 | 1.57E-02 |
| ko00030 | Pentose phosphate pathway | 8 | 47 | 2.55E-02 |
| ko05020 | Prion diseases | 9 | 60 | 3.00E-02 |
| ko04060 | Cytokine-cytokine receptor interaction | 37 | 469 | 3.15E-02 |
| ko04978 | Mineral absorption | 12 | 97 | 3.15E-02 |
| ko04974 | Protein digestion and absorption | 21 | 228 | 4.10E-02 |
| ko04976 | Bile secretion | 16 | 159 | 4.44E-02 |
| ko05144 | Malaria | 11 | 91 | 4.44E-02 |

**Table S4. List of selected mRNAs, grouped according to functional classes (shown in bold), found to be up- and down-regulated by buccal infection with Ich (14 d and 28 d). Related to Figure 3**

## Transparent Methods

### Fish maintenance

**Adult rainbow trout (triploid female fish, mean weight = 200–300 g) used for oral bacteria isolation and routine histology and juvenile rainbow trout (triploid female fish, mean weight = 20-30 g) used in infection trials were obtained from a fish farm in Shiyan (Hubei, China),** and maintained in aquarium tanks with a water recirculation system including thermostatic temperature control and extensive biofiltration. Fish were acclimatized for at least 2 weeks at 15 ℃ and fed daily with commercial trout pellets at a rate of 0.5–1% biomass during the whole experiment periods. The feeding was terminated 48 h prior sacrifice. Japanese sea bass (*Lateolabrax japonicus*), Asian swamp eel (*Monopterus albus*), Southern catfish (*Silurus meridionalis*) and Snakehead (*Channa argus*) were purchased from aquatic product market in Wuhan (Hubei, China). Animal procedures were approved by the Animal Experiment Committee of Huazhong Agricultural University.

### Ich parasite isolation and infection

For Ich parasite isolation, the method was described previously with slight modification (Yu et al., 2018). Briefly, heavily infected rainbow trout were anaesthetized with an overdose of MS-222 and placed in a beaker with water to allow trophonts and tomonts to exit the host. Fish were removed 4 h later, while the trophonts and tomonts were left in the water at 15 ℃ for 24 h to allow tomocyst formation and subsequent theront release. For Ich infection, two types of challenges were performed. In the first group, fish were exposed to a single dose of ~5,000 theronts per fish for 3 hours, and then migrated into the aquarium containing new aquatic water. Tissue samples including BM, head kidney and spleen were taken 0.5, 1, 4, 7, 14, 21, 28, and 75 days after infection. Moreover, fluids (serum and buccal mucus) were taken after 28 days (infected fish). In the second group, fish were monthly exposed for 75 days period (survivor fish) with the same dose. Fish samples were taken two weeks

after the last challenge. Both experiments were performed at least three independent times. As a control (mock infected), the same number of fishes were maintained in a similar tank but without parasites.

**Collection of serum, buccal mucus and bacteria**

**For sampling, trout were anesthetized with MS-222, and serum was collected by centrifugation for 10 min at 4 ℃, 5000 *g* and stored at -80 ℃ prior to use** (Xu et al., 2016). To obtain the buccal mucus, briefly, fish BM tissue was excised and rinsed with PBS three times to remove the remaining blood. Thereafter BM tissue was incubated for 12 h at 4 ℃, with slightly shaking in protease inhibitor buffer (1× PBS, containing 1× protease inhibitor cocktail [Roche], 1 mM phenylmethylsulfony fluoride [Sigma]; pH 7.2) at a ratio of 250 mg of BM tissue per mL of buffer. The suspension (buccal mucus) was collected into an Eppendorf tube, and then vigorously vortexed and centrifuged at 400 *g* for 10 min at 4 ℃ to remove trout cells. To separate buccal bacteria from mucus, the cell-free supernatant was centrifuged at 10,000 *g* for 10 min at 4 ℃. The resulting supernatant (containing buccal mucus) was harvested, filtered with 0.45 μm syringe filter (Millipore) and stored at 4 ℃ prior to use. The pellet (containing buccal bacteria) was washed three times with 1 mL of cold PBS (pH 7.2) and resuspended for further analysis.

**Gel filtration**

Gel filtration were performed to analyze the monomeric or polymeric state of Igs in trout buccal mucus using a Superdex-200 FPLC column (GE Healthcare) as presented previously for gut mucus (Zhang et al., 2010). The column was previously equilibrated with cold PBS (pH 7.2), and protein fractions were eluted at 0.5 mL/min with PBS using a fast protein LC instrument with ÄKTApurifier systems (GE Healthcare). Identification of IgM, IgD and IgT in the eluted fractions was performed by western blot using anti-IgM, anti-IgD and anti-IgT antibodies, respectively. A standard curve was generated by plotting the elution volume of the standard proteins in a Gel Filtration Standard (Bio-

Rad) against their known molecular weight, which was then used to determine the molecular weight of the eluted IgT, IgM and IgD by their elution volume.

**Isolation of trout head kidney and BM leucocytes**

**To isolate trout head kidney and BM leucocytes, we modified the existing protocol as explained by us (Yu et al., 2018).** Briefly, we anaesthetized the rainbow trout with MS-222 and collected the blood from the caudal vein. Trout head kidneys were removed aseptically and pressed through a 100-μm nylon mesh and suspended in Dulbecco's modified eagle medium (DMEM, supplemented with 5% FBS, 100 U/mL penicillin and 100 μg/mL streptomycin). Then the BM was taken and washed with cold PBS to avoid blood contamination. Thereafter, the BM was cut into small pieces (approximately 0.1 $cm^2$) in DMEM and then mechanically disaggregated on a 100-μm cell shredder on the ice. The cell fraction was collected, and the aforementioned procedure was repeated four times. The non-disaggregated BM tissue pieces were treated with PBS (containing 0.37 mg/mL EDTA and 0.14 mg/mL dithiothreitol DTT) for 30 min followed by enzymatic digestion with collagenase (Invitrogen, 0.15 mg/mL in PBS) for 30 h at 20 ℃ with continuous shaking, and mechanically disaggregated on a 100-μm cell shredder and the cell fraction was collected. Subsequently, the cell fractions from the above BM tissue treatments were pooled and passed through a 100-μm nylon mesh. Finally, the resulting cell fractions were washed three times in fresh DMEM and layered over a 51/34% discontinuous Percoll gradient. After 30 min of centrifugation at 400 *g*, cells lying at the interface of the gradient were collected and washed with DMEM medium.

**SDS-PAGE and western blot**

Serum and buccal mucus samples were resolved on 4–15% SDS-PAGE Ready Gel (Bio-Rad) under non-reducing and/or reducing conditions. For western blot analysis, the gels were transferred onto PVDF membranes (Bio-Rad). Thereafter, the membranes were blocked with 8% skim milk and

incubated with anti-trout IgT (rabbit polyclone antibody [pAb]) anti-trout IgM (mouse monoclonal antibody [mAb]) or biotinylated anti-trout IgD (mouse mAb) antibodies followed by incubation with peroxidase-conjugated anti-rabbit, anti-mouse IgG (Invitrogen) or streptavidin (Invitrogen). Immunoreactivity was detected with an enhanced chemiluminescent reagent (Advansta) and scanned by GE Amersham Imager 600 Imaging System (GE Healthcare). The captured gel images were analyzed by ImageQuant TL software (GE Healthcare). Thereafter, the concentration of IgM, IgD and IgT were determined by plotting the obtained signal strength values on a standard curve generated for each blot using known amounts of purified trout IgM, IgD or IgT.

**Flow cytometry**

For flow cytometry analysis, leukocytes suspensions of trout head kidney and BM were double-stained with monoclonal mouse anti-trout IgT and anti-trout IgM (1 μg/mL each) on ice for 45 min. After washing three times, APC-goat anti-mouse IgG2b and PE-goat anti-mouse IgG1 (1 μg/mL each, BD Biosciences) were added and incubated for 45 min at 4 ℃ to detect $IgT^+$ and $IgM^+$ B-cells, respectively. Buccal bacteria were stained with mouse anti-trout IgM (1 μg/mL), anti-trout IgD (1 μg/mL), anti-trout IgT (2 μg/mL) or their respective isotype controls (1 μg/mL) at 4 °C for 1 h with continuous agitation. After washing three times, Alexa Fluor 488-goat anti-mouse IgG1, and Alexa Fluor 488-goat anti-mouse IgG2b were added respectively, and incubated for 1 h at 4 ℃. To discriminate bacteria from debris, buccal bacteria were labelled with BacLight Red bacterial stain (Invitrogen), following the manufacturer’s instructions. After washing three times, analysis of stained leucocytes or bacteria was performed with a CytoFLEX flow cytometer (Beckman coulter) and analyzed by FlowJo software (Tree Star).

**Histology, light microscopy and immunofluorescence microscopy studies**

The BMs of control adult rainbow trout, southern catfish, Japanese seabass, and Chinese sturgeon, as well as the infected rainbow trout was dissected and processed for routine histology. All the BMs were fixed in 4% neutral buffered formalin, embedded in paraffin, sectioned, and stained with H&E and AB (Yu et al., 2018; Yashpal et al., 2007). Images were acquired in microscope (Olympus) using the Axiovision software. For the detection of Ich parasite as well as $IgT^+$ and $IgM^+$ B-cells, sections were stained with polyclonal rabbit anti-trout IgT (pAb; 0.49 μg/mL) and monoclonal mouse anti-trout IgM (IgG1 isotype; 1 μg/mL) for 2 h at 37 ℃. After washing three times, sections were stained with Alexa Fluor 488-conjugated AffiniPure Goat anti-rabbit IgG and Cy3-conjugated AffiniPure Goat anti-mouse IgG (Jackson ImmunoResearch Laboratories Inc.) at 2.5 μg/mL each for 40 min at room temperature to detect $IgT^+$ and $IgM^+$ B-cells, respectively. After washing three times with PBS, mouse anti-Ich polyclonal antibody (1 μg/mL) was added and incubated at 4 °C for 6 h. After washing three times, Alexa Fluor 647-goat anti-mouse IgG (Jackson ImmunoResearch Laboratories Inc.) with 5 μg/mL were added and incubated at room temperature for 40 min to detect Ich parasite. For detection of trout buccal pIgR, we used the same methodology described to stain trout skin pIgR by using our rabbit anti-pIgR antibody (Zhang et al., 2010). As isotype controls, the rabbit IgG prebleed and the mouse-IgG1 isotype antibodies were labelled with the same antibody labelling kits and used at the same concentrations. All sections were stained with DAPI (4', 6-diamidino-2-phenylindole; 1 μg/mL: Invitrogen) before mounting. For visualization of coating of buccal bacteria with IgT, IgM and IgD, the bacteria were firstly double-stained with rabbit anti-trout IgT and mouse anti-trout IgM (1 μg/mL each), or isotype controls (the rabbit IgG and the mouse-IgG1 (1 μg/mL each) at 4 ℃ for 2 h with continuous agitation. After washing three times, the secondary antibodies Alexa Fluor 488-conjugated AffiniPure Goat anti-rabbit IgG or Cy3-conjugated AffiniPure Goat anti-mouse IgG (Jackson ImmunoResearch Laboratories Inc.) at 2.5 μg/mL each were added and

incubated for 30 min at 4 ℃. After washing as described above, biotin-labelled mouse anti-IgD antibody (1 μg/mL) was added and incubated at 4 ℃ for 2 h, after washing three times, Alexa Fluor 647-conjugated Streptavidin (Jackson ImmunoResearch Laboratories Inc.) with 5 μg/mL were added and incubated at 4 ℃ for 30 min. Before mounting, bacteria were stained with a mixed solution of DAPI and Hoechst 33342 dye (5 μg/mL; Molecular Probes). Stained bacteria were cytospinned on glass slides and mounted with fluorescent microscopy mounting solution. All images were acquired and analyzed using an Olympus BX53 fluorescence microscope (Olympus) and the iVision-Mac scientific imaging processing software (Olympus).

**Proliferation of B-cells in the BM of trout**

For the proliferation of B-cells studies, we modified the methodology as previously reported by us (Xu et al., 2016; Yu et al., 2018). Briefly, control and survivor fish (~30 g) were anaesthetized with MS-222 and intravenous injected with 200 μg EdU (Invitrogen). After 24 h, leucocytes from BM and head kidney were isolated as described above. Subsequently, cells were incubated with mAb mouse anti-trout IgM and anti-trout IgT (1 μg/mL each) on ice for 1 h. After washing three times with DMEM medium, Alexa Fluor 488-goat anti-mouse IgG (Invitrogen) was used as secondary antibody to detect $IgM^+$ or $IgT^+$ B-cells. After 30 min incubation on ice, cells were washed three times and then fixed with 4% neutral buffered formalin for 15 min. $EdU^+$ cell detection was performed according to the manufacturer's instructions (Click-iT EdU Alexa Fluor 647 Flow Cytometry Assay Kit, Invitrogen). Cells were thereafter analyzed in a CytoFLEX flow cytometer (Beckman coulter) and FlowJo software (Tree Star). For immunofluorescence analysis, the paraffin sections of BM were incubated with rabbit anti-trout IgT (pAb; 1 μg/mL) and mouse anti-trout IgM (IgG1 isotype; 1 μg/mL) at 4°C for 45 min. After washing with PBS, paraffin sections were incubated with Alexa Fluor 488-conjugated AffiniPure Goat anti-rabbit IgG or Cy3-conjugated AffiniPure Goat anti-

mouse IgG (Jackson ImmunoResearch Laboratories Inc.) at 2.5 μg/mL each at room temperature for 45 min. Stained cells were fixed with 4% neutral buffered formalin and $EdU^+$ cell detection was performed according to the manufacturer's instructions (Click-iT EdU Alexa Fluor 647 Imaging Kit, Invitrogen). Cell nuclei were stained with DAPI (1 μg/mL) before mounting with fluorescent microscopy mounting solution. Images were acquired and analyzed using an Olympus BX53 fluorescence microscope (Olympus) and the iVision-Mac scientific imaging processing software (Olympus).

**Tissue explants culture**

Control and survivor fish were anaesthetized with an overdose of MS-222, and blood was removed from the caudal vein to avoid blood content in the collected organs. Thereafter, spleen, head kidney, and BM were collected. Approximately 20 mg of each tissue was isolated and submerged in 70% ethanol for 1 min to eliminate possible bacteria on their surface and then washed twice with PBS. Subsequently, tissues were transferred into a 24-well plate and cultured with 200 μL DMEM medium (Invitrogen), supplemented with 10% FBS, 100 U/mL penicillin, 100 μg/mL streptomycin, 200 μg/mL amphotericin B and 250 μg/mL gentamycin sulfate, with 5% $CO_2$ at 17 ℃. After 7 days, supernatants were harvested, centrifuged and stored at 4 ℃ prior to use at the same day.

**Binding of trout immunoglobulins to Ich**

To assess whether infected and survivor fish had generated pathogen-specific immunoglobulins, we measured the capacity of IgT, IgM and IgD from serum, buccal mucus or tissue (BM, spleen and head kidney) explant supernatants to bind to Ich using a pull-down assay as described previously (Yu et al., 2018). Initially, approximately 100 tomonts were pre-incubated with a solution of 0.5% BSA in PBS (pH 7.2) at 4 ℃ for 2 h. Subsequently, tomonts were incubated with diluted fluids samples (buccal mucus, serum, or tissue explant supernatants) separately from infected, survivor, or control

fish at 4 °C for 4 h with continuous shaking in a 300 μL volume. After incubation, the tomonts were washed three times with PBS and bound proteins were eluted with 2 × Laemmli Sample Buffer (Bio-Rad) and boiled for 5 min at 95 ℃. The eluted material was resolved on 4–15% SDS-PAGE Ready Gel under non-reducing conditions, and the presence of IgT, IgM or IgD was detected by western blotting using the anti-trout IgT, IgM or IgD antibody as described above.

**Co-immunoprecipitation studies**

We followed the same strategy to detect the association of pIgR to IgT in gill mucus as we previously described (Xu et al., 2016). To detect whether polymeric trout IgT present in the buccal mucus were associated to a secretory component-like molecule derived from tSC, we performed co-immunoprecipitation analysis using anti-trout IgT (pAb) antibody with the goal to potentially co-immunoprecipitate the secretory component of trout (tSC). To this end, 10 μg of anti-IgT antibody were incubated with 100 μL of trout buccal mucus. As control for these studies, the same amount of rabbit control IgG (purified from the prebleed serum of the rabbit) were used as negative controls for anti-IgT. After overnight incubation at 4 °C, 20 μL of protein G Agarose (Invitrogen) was added into each reaction mixture and incubated for 1 hour at 4 ℃. Thereafter, the beads were washed five times with cold PBS, and subsequently bound proteins eluted in 2 × Laemmli Sample Buffer (Bio-Rad). The eluted material was resolved by SDS-PAGE on 4–15% Tris-HCl Gradient ReadyGels (Bio-Rad) under reducing (for tSC detection) or non-reducing (for IgT detection) conditions. Western blot was performed with anti-pIgR or anti-IgT antibody as described above.

**RNA isolation and quantitative real-time PCR (qPCR) analysis**

Total RNA was extracted by homogenization in 1 mL TRIZol (Invitrogen) using steel beads and shaking (60 HZ for 1 min) following the manufacturer's instructions. A spectrophotometry (NanoPhotometer NP 80 Touch) was used to quantitate the extracted RNA and agarose gel

electrophoresis was used to determine the integrity of the RNA. To normalize gene expression levels, equivalent amounts of the total RNA (1000 ng) of each sample were used for cDNA synthesis with the SuperScript first-strand synthesis system for qPCR (Yeasen) in a 20 μL reaction volume. The synthesized cDNA was diluted 4 times and then used as a template for qPCR analysis. The qPCRs were performed on a 7500 qPCR system (Applied Biosystems) using the EvaGreen 2× qPCR Master mix (Yeasen). All samples were performed following conditions: 95 °C for 5 min, followed by 40 cycles at 95 °C for 10 s and at 58 °C for 30 s. A dissociation protocol was carried out after thermos cycling to confirm a band of the correct size was amplified. Trout housekeeping gene elongation factor 1α (EF1α) was used as control gene for normalization of expression. Primer sequences can be found in Table S1. The relative expression level of the genes was determined using the Pfaffl method (Pfaffl et al., 2001).

**RNA-Seq libraries and RNA-seq analysis**

The RNA-Seq libraries from twelve samples were generated according as a previous study (Abyzov et al., 2012). Briefly, polyadenylated RNA fragments were purified by a Dynabeads mRNA Purification Kit, fragmented with RNA fragmentation buffer, and reverse transcribed into first-strand cDNA using random hexamer and Superscript II reverse transcriptase, followed by second-strand cDNA synthesis using RNaseH and DNA polymerase I. The resulting cDNA was end-repaired, and a single "A" was added at the 3' ends and a unique identifier (UID) was labelled at the 5' ends before ligating to Illumina paired-end sequencing adaptors. PCR-amplified using Phusion High-Fidelity master mix and Illumina primers with the condition of 98 ℃ for 60 s, 15 cycles of 98 ℃ for 10 s, and 65 ℃ for 75 s, and concluding with 65 ℃ for 5 min.

All RNA-seq data were generated by Illumina paired-end sequencing with read length 150 bp. Reads were mapped to the *Oncorhynchus mykiss* genome using STAR with default parameters

(Dobin et al., 2013). The mapped reads were analyzed by featureCounts (Liao et al., 2014). Differential expression was estimated with edgeR package (Robinson et al., 2010). We excluded the genes with low expression (CPM [count-per-million] < 1 in nine or more samples) from downstream analysis. The resulting genes were considered as differentially expressed genes (DEGs) if FDR ≤ 0.05 and $|\log_2$ (fold-change) $| \geq 1$. Pathway analysis of significantly differential expressed genes was conducted with DAVID using all the expressed genes as background (Huang et al., 2009).

**Statistics**

An unpaired Student's *t*-test and one-way ANOVA with Bonferroni correction (Prism version 6.0; GraphPad) were used for analysis of differences between groups. p values of 0.05 or less were considered statistically significant.

**Availability of data and material**

The NCBI Sequence Read Archive (SRA) accession number for the data reported in this manuscript is PRJNA560142.

## SUPPLEMENTAL REFERENCES


Abyzov, A., Mariani, J., Palejev, D., Zhang, Y., Haney, M.S., Tomasini, L., Ferrandino, A.F., Rosenberg Belmaker, L.A., Szekely, A., Wilson, M., et al. (2012). Somatic copy number mosaicism in human skin revealed by induced pluripotent stem cells. Nature *492*, 438.

Dobin, A., Davis, C.A., Schlesinger, F., Drenkow, J., Zaleski, C., Jha, S., Batut, P., Chaisson, M., and Gingeras, T.R. (2013). STAR: ultrafast universal RNA-seq aligner. Bioinformatics *29*, 15–21.

Huang da, W., Sherman, B.T., and Lempicki, R.A. (2009). Bioinformatics enrichment tools: paths toward the comprehensive functional analysis of large gene lists. Nucleic Acids Res. *37*, 1–13.

Liao, Y., Smyth, G.K., Shi, W. (2014). feature Counts: an efficientgeneral-purpose program forassigning sequence reads togenomic features. Bioinformatics *30*, 923–930.

Pfaffl, M.W. (2001). A new mathematical model for relative quantification in real-time RT-PCR. Nucleic Acids Res. *29*, e45.

Robinson, M.D., McCarthy, D.J., Smyth, G.K. (2010). "edgeR: a Bioconductor package for differential expression analysis of digital gene expression data." Bioinformatics *26*, 139–140.

Xu, Z., Takizawa, F., Parra, D., Gomez, D., von Gersdorff Jorgensen, L., LaPatra, S.E., and Sunyer, J.O. (2016). Mucosal immunoglobulins at respiratory surfaces mark an ancient association that predates the emergence of tetrapods. Nat. Commun. *7*, 10728.

Yashpal, M., Kumari, U., Mittal, S., and Mittal, A.K. (2007). Histochemical characterization of glycoproteins in the buccal epithelium of the catfish, Rita rita. Acta. Histochem. *109*, 285–303.

Yu, Y.Y., Kong, W.G., Yin, Y.X., Dong, F., Huang, Z.Y., Yin, G.M., Dong, S., Salinas, I., Zhang, Y.A., Xu, Z. (2018). Mucosal immunoglobulins protect the olfactory organ of teleost fish against parasitic infection. PLoS Pathog. *14*, e1007251.

Zhang, Y.A., Salinas, I., Li, J., Parra, D., Bjork, S., Xu, Z., LaPatra, S.E., Bartholomew, J., and Sunyer, J.O. (2010). IgT, a primitive immunoglobulin class specialized in mucosal immunity. Nat. Immunol. *11*, 827–835.
